# Across-breed genetic investigation of canine hip dysplasia, elbow dysplasia, and anterior cruciate ligament rupture using whole-genome sequencing

**DOI:** 10.3389/fgene.2022.913354

**Published:** 2022-12-02

**Authors:** Emily E. Binversie, Mehdi Momen, Guilherme J. M. Rosa, Brian W. Davis, Peter Muir

**Affiliations:** ^1^ Comparative Orthopaedic and Genetics Research Laboratory, School of Veterinary Medicine, University of Wisconsin-Madison, Madison, WI, United States; ^2^ Department of Animal and Dairy Sciences, College of Agricultural and Life Sciences, University of Wisconsin-Madison, Madison, WI, United States; ^3^ Department of Veterinary Integrative Biosciences, College of Veterinary Medicine and Biomedical Sciences, Texas A&M University, College Station, TX, United States

**Keywords:** dog, GWAS, breed phenotypes, whole-genome sequencing SNPs, spontaneous orthopedic disease, weighted least squares regression

## Abstract

Here, we report the use of genome-wide association study (GWAS) for the analysis of canine whole-genome sequencing (WGS) repository data using breed phenotypes. Single-nucleotide polymorphisms (SNPs) were called from WGS data from 648 dogs that included 119 breeds from the Dog10K Genomes Project. Next, we assigned breed phenotypes for hip dysplasia (Orthopedic Foundation for Animals (OFA) HD, *n* = 230 dogs from 27 breeds; hospital HD, *n* = 279 dogs from 38 breeds), elbow dysplasia (ED, *n* = 230 dogs from 27 breeds), and anterior cruciate ligament rupture (ACL rupture, *n* = 279 dogs from 38 breeds), the three most important canine spontaneous complex orthopedic diseases. Substantial morbidity is common with these diseases. Previous within- and between-breed GWAS for HD, ED, and ACL rupture using array SNPs have identified disease-associated loci. Individual disease phenotypes are lacking in repository data. There is a critical knowledge gap regarding the optimal approach to undertake categorical GWAS without individual phenotypes. We considered four GWAS approaches: a classical linear mixed model, a haplotype-based model, a binary case-control model, and a weighted least squares model using SNP average allelic frequency. We found that categorical GWAS was able to validate HD candidate loci. Additionally, we discovered novel candidate loci and genes for all three diseases, including *FBX025, IL1A, IL1B, COL27A1, SPRED2* (HD), *UGDH*, *FAF1* (ED), *TGIF2* (ED & ACL rupture), and *IL22*, *IL26*, *CSMD1*, *LDHA*, and *TNS1* (ACL rupture). Therefore, categorical GWAS of ancestral dog populations may contribute to the understanding of any disease for which breed epidemiological risk data are available, including diseases for which GWAS has not been performed and candidate loci remain elusive.

## Introduction

In recent years, organized efforts have assembled large repositories of whole-genome sequencing (WGS) data, such as the international Dog10K Consortium (Ostrander et al., 2019). The Dog10K Consortium aims to generate ∼20x coverage WGS data from 10,000 dogs and use this large dataset to investigate domestication, breed formation, aging, behavior, and morphologic variation. Breed and sex are the only phenotypic data recorded. The number of dogs from each breed in the repository varies from a few individuals to more than 50 dogs. To maximize the use of WGS repository data for the investigation of canine polygenic disease, new statistical approaches are needed for the analysis of breed phenotypes with an unbalanced number of individuals per breed.

In dogs, breed phenotypes rather than individual case-control or quantitative phenotypes have been used to study height, chondrodysplasia, body size, muscling, skull shape, ear type, snout length, fur length and quality, life expectancy, cognition, behavior, cesarean section rate, litter size, stillbirth rate, gestation length, and cancer mortality (Jones et al., 2008; Sutter et al., 2008; Parker et al., 2009; Bannasch et al., 2010; Boyko et al., 2010; Vaysse et al., 2011; Hoopes et al., 2012; Schoenebeck et al., 2012; Hayward et al., 2016; Plassais et al., 2017; Plassais et al., 2019; Smith et al., 2019; Bannasch et al., 2020; Doherty et al., 2020; Gnanadesikan et al., 2020). Domestication has produced dog breeds with substantial phenotypic variation. Weight and height can vary substantially (Supplementary Figure S1). Over 400 breeds exist, defined by behavioral and physical characteristics. Strong artificial selection and population bottlenecks have resulted in breeds with fixed phenotypes, such as chondrodysplasia (Parker et al., 2017).

Disease risk variants have undergone inadvertent selection within breeds due to genetic correlation caused by random fixation, linkage disequilibrium (LD), and pleiotropy (Supplementary Figure S2). High disease prevalence in a breed reflects the enrichment of disease risk alleles during modern breed creation and domestication (Sargan, 2004; Karlsson and Lindblad-Toh 2008). Disease risk variants overrepresented within a breed allow genome-wide association study (GWAS) to be performed using breed phenotypes for the discovery of large effect variants associated with reproductive traits (Smith et al., 2019), longevity (Plassais et al., 2019; Doherty et al., 2020), and cancer (Doherty et al., 2020).

Although single-nucleotide polymorphism (SNP) array data can detect some fixed large-effect genetic variants, such data are underpowered compared to WGS SNPs for the discovery of all potential associations that may exist across many breeds (Hayward et al., 2016). Genetic risk for many complex diseases is also influenced by numerous small to moderate effect loci responsible for within-breed heterogeneity (Supplementary Figure S3) (Hayward et al., 2016; Bannasch et al., 2020). Within a breed, heterogeneity is best captured by within-breed GWAS with individual-level phenotypes (Hayward et al., 2016). Disease-associated variants with lesser effects that are responsible for within-breed phenotypic heterogeneity are not captured by across-breed GWAS with breed phenotypes. Across-breed GWAS with WGS SNPs and breed phenotypes is a useful approach to discover fixed large-effect variants that are shared across breeds that would otherwise go undiscovered. However, this approach has not been investigated as an approach for the genetic discovery of common complex diseases using repository data.

Previous research has used a classical linear mixed model (LMM) approach and removed individuals from overrepresented breeds to balance breed representation (Plassais et al., 2019; Doherty et al., 2020). However, LMMs do not properly support multilevel categorical phenotypes. For this study, we developed a new statistical approach for analyzing repository sequence data with breed phenotypes and unbalanced breed representation. We performed the first GWAS using WGS SNPs and breed phenotypes to discover large-effect genetic variants shared across breeds for the three major economically important canine orthopedic diseases, namely hip dysplasia (HD), elbow dysplasia (ED), and anterior cruciate ligament (ACL) rupture (Johnson et al., 1994; Wilke et al., 2005; Michelsen, 2013). HD, ED, and ACL rupture are complex polygenic diseases with moderate heritability, where disease risk varies considerably between breeds (Witsberger et al., 2008; Oberbauer et al., 2017). Heritability estimates of HD range between 0.1–0.6 (Fries and Remedios 1995; Ginja et al., 2009; Krotscheck and Todhunter 2010; Lewis et al., 2013). For ED, heritability estimates range from 0.10–0.77 (Guthrie and Pidduck, 1990; Beuing et al., 2000; Mäki et al., 2000; Soo et al., 2018) and for ACL rupture, heritability estimates range from 0.27–0.89 (Nielen et al., 2001; Wilke et al., 2006; Baker et al., 2017; Cook et al., 2020). Previous within- and between-breed GWAS for HD, ED, and ACL rupture have identified candidate loci using array SNPs (Pfahler and Distl 2012; Baird et al., 2014; Hayward et al., 2016; Baker et al., 2017; Mikkola et al., 2019). Studies investigating HD and ACL rupture have found associations in genes influencing morphology (Fealey et al., 2017; Baker et al., 2018; Healey et al., 2019; Baker et al., 2021). The discovery of shared across-breed genetic variants for these diseases has not been previously undertaken.

To study WGS SNPs using breed phenotypes and unbalanced breed representation, we used four association models for GWAS of HD, ED, and ACL rupture. We were able to detect new biologically relevant ED associations and validate previously reported associations for HD. We also report HD’s association with genes that have been under selection since domestication. These domestication genes influence traits such as body size, ear phenotype, and behavior. This suggests that HD is a complex trait that may have been directly or indirectly influenced by the selection of morphological traits during canine domestication.

## Materials and methods

### Whole-genome sequencing samples

High-depth WGS data from 648 canids, including 562 dogs from 119 breeds, were obtained from the Dog10K Consortium (Ostrander et al., 2019). Dog10K performed alignment to CanFam3.1 and called 45,165,129 variants (Ostrander et al., 2019). Variant mean depth, variant missingness, variant quality, individual mean depth, and individual missingness analyses were performed using vcftools (Danecek et al., 2011) and tidyverse in R (Wickham et al., 2019).

### Breed filtering

In an across-breed GWAS with breed phenotypes, it is critical that breed assignments are correct. To detect samples with inaccurate breed assignment, we performed neighbor-joining phylogeny analysis. We compared the 562 dogs with a breed assignment to a previously published dataset of 1,110 dogs from 129 breeds with SNP array data (accession: GSE123368, GSE70454, GSE83160, GSE90441, and GSE96736) (Vaysse et al., 2011; Decker et al., 2015; Dreger et al., 2016a; 2016b; Parker et al., 2017; Plassais et al., 2019). VCFtools was used to filter WGS SNPs to retain biallelic SNPs, remove indels, and remove variants with a quality score under 10 (Danecek et al., 2011). SNP locations were extracted from WGS data and merged with SNP array data. 143,706 SNPs were used to calculate 100 bootstrapped pairwise identity-by-state (IBS) distance matrices using PLINK with the –genome and –cluster commands (Purcell et al., 2007; Parker et al., 2017). The neighbor program in PHYLIP drew 100 trees from the bootstrapped IBS matrices (Rambaut 2006; Parker et al., 2017). The consensus program in PHYLIP then combined the 100 trees to build a consensus tree by majority rule (Felsenstein, 1989). FigTree v1.4.4 was used to plot the consensus tree (Rambaut 2006). Any of the 562 dogs with WGS SNPs that did not cluster within their breed as expected were excluded from subsequent analysis.

### Breed phenotypes for hip dysplasia, elbow dysplasia, and anterior cruciate ligament rupture

Breed phenotypes for HD, ED, and ACL rupture were assigned based on epidemiological data describing disease prevalence (Witsberger et al., 2008; Oberbauer et al., 2017). Because both publications reported HD prevalence for different breeds and used different methods, we analyzed them separately (Orthopedic Foundation for Animals (OFA) HD prevalence (Oberbauer et al., 2017) and hospital HD prevalence from 27 veterinary teaching hospitals in North America from 1964 to 2003 (Witsberger et al., 2008)).

### Individual and variant filtering

VCFtools was used to retain biallelic SNPs, remove indels, and remove variants with a quality score under 20 (Danecek et al., 2011). The quality score threshold of 20 was selected based on earlier work (Plassais et al., 2019). Dogs within the filtered Dog10K dataset were retained if that breed had a reported prevalence for HD, ED, or ACL rupture. Samples and SNPs were then filtered in PLINK (Purcell et al., 2007). Dogs were removed if they had a genotyping rate ≤ 90%. SNPs were removed if the genotyping rate was ≤ 99%, minor allele frequency (MAF) was ≤ 0.05, or they were heterozygous haploid calls. Hardy–Weinberg test statistics were generated for each SNP. After SNP filtering, 7,586,942 SNPs were in the OFA HD and OFA ED SNP sets, and 7,026,774 SNPs were in the hospital HD and ACL rupture SNP sets.

### Literature search for loci associated with hip dysplasia, elbow dysplasia, and anterior cruciate ligament rupture

A literature search was performed to create a comprehensive list of all previously reported associated loci with HD, ED, and ACL rupture in dogs.

### Genome-wide association study

#### Linear mixed model

A centered relatedness matrix was calculated using GEMMA v0.94.1 for classical LMM association analysis with sex as a covariate (Zhou and Stephens 2012). The Wald test was used to determine *P* values, and Bonferroni correction (0.05/number of variants) was used to identify significant associations for OFA HD and ED (7,586,942 SNPs) (*p* < 6.59E-9) (Oberbauer et al., 2017) and hospital HD and ACL rupture (7,026,774 SNPs) (*p* < 7.12E-9) (Witsberger et al., 2008). All Quantile–Quantile (QQ) and Manhattan plots were constructed using R. For comparison to Bonferroni correction of LMM results, a separate false discovery rate (FDR) correction of *P* values used the fdrtool in R (Strimmer 2008).

#### Binary case-control model

A binary association was performed by selecting a group of breeds that are strongly protected from the trait (low-risk controls) and a group of breeds that are strongly predisposed (high-risk cases) for the association. Two different scenarios were compared. Initially, we used a stringent scenario with a full separation of cases and controls to ensure the prevalence cutoffs were disparate. Since a limited number of dogs from low- and high-risk breeds for HD, ED, and ACL rupture were available, we also tested a less stringent scenario where the threshold values produced case and control groups with a larger sample group size. The case-control binary association was performed using GEMMA with a genomic relationship matrix and sex as a covariate (Zhou and Stephens 2012). As described in the GEMMA software, all of the controls are regarded as zero, while all of the cases are regarded as one. When the SNP effect size is small, this approximation is relatively accurate (Zhou et al., 2013). The Wald test was used to determine *P* values, and Bonferroni correction was used to identify significant associations for OFA HD and ED (stringent binary case-control *p* < 6.68E-9) (lenient binary case-control *p* < 6.64E-9) (Oberbauer et al., 2017) and hospital HD and ACL rupture (stringent binary case-control *p* < 7.26E-9) (lenient binary case-control *p* < 7.13E-9) (Witsberger et al., 2008).

#### Haplotype-based model

Fixed width haplotype-based association was performed with PLINK (Purcell et al., 2007). A fixed 4 SNP window was selected based on a preliminary comparison of differing window sizes. The centered relatedness matrices previously generated were used to draw scree plots in R to determine the optimum number of eigenvectors to correct for the population structure after assessment using Catell’s scree test (Cangelosi and Goriely 2007). For the OFA HD and ED datasets, the first six eigenvectors and for the hospital HD and ACL rupture datasets the first eight eigenvectors were incorporated as covariates. When the haplotype-based analysis was performed with a fixed 4 SNP window, 7,586,822 and 7,026,651 sliding windows were analyzed for the OFA (HD and ED) and veterinary hospital (HD and ED) datasets, respectively. A Bonferroni correction was used to identify significant associations for OFA HD and ED (*p* < 1.68E-9) (Oberbauer et al., 2017) and hospital HD and ACL rupture (*p* < 1.82E-9) (Witsberger et al., 2008). For comparison to Bonferroni correction of haplotype-based results, a separate FDR correction of *P* values was performed with fdrtool in R (Strimmer 2008).

#### Weighted least squares model

We used a simple linear regression model with weights on the averaged genotypes as our final approach to perform categorical GWAS with breed phenotypes. Initially, we computed the average of the SNP dosage for each locus in each dog breed. So, we had an X matrix (n*p) for each disease phenotype where n = number of dog breeds and p = number of SNPs. We then computed the principal components from the genomic relationship matrix and included the first five important principal components in the model. For each breed, the weight was the proportion of the genotyped individuals for each breed in the whole sample population. Finally, we ran the following model and estimated SNP effects in the R environment:

lm(y ∼ pc1+pc2+pc3+pc4+pc5+snp_j, data=X, weights=Wt),

where X = average SNP dosage matrix, y = disease prevalence in the breed, and Wt = proportion of individuals of each breed in the GWAS sample.

For each SNP within a breed, an SNP average allelic frequency was calculated. Because the confidence that the SNP average allelic frequency appropriately represents the true breed average is related to the number of individual dogs within a breed, the GWAS was weighted according to the number of individuals (repeats) within each breed. This model has the advantage of using all the SNP data and appropriately considers variation in the number of dogs in each breed. A Bonferroni correction was used to identify significant associations for OFA HD and ED (*p* < 6.59E-9) (Oberbauer et al., 2017) and hospital HD and ACL rupture (*p* < 7.11E-9) (Witsberger et al., 2008).

### Gene annotation

The CanFam3.1 build on the UCSC and Ensembl genome browsers was used to identify genes contained within the top candidate loci. Due to the increased density of the WGS SNPs no flanking regions were added for gene annotation.

## Results

### Whole-genome sequencing, breed, and SNP filtering

Initial assessment of the unfiltered Dog10K variants from 648 canids (*n* = 5 wolves, *n* = 33 village/indigenous dogs, *n* = 562 domestic dogs (119 breeds), *n* = 14 mixed dogs/breeds, and *n* = 34 dogs of unknown breed) was performed (Supplementary Table S1, Supplementary Figure S4). Variant mean depth ranged from 0 to 1,541.1, with a mean of 18.8. Variant missingness ranged from 0.15% to 99.85%, with a mean of 7.54%. Variant quality ranged from 30 to 25,177,600, with a mean of 43,170. The minimum mean depth of the samples was 0.01, with a mean of 18.88 and a maximum of 43.19. The percent missingness of the samples ranged from 2.23% to 99.88%, with a mean of 7.54%.

Breed assignment of purebred dogs in the Dog10K dataset was verified by performing neighbor-joining phylogeny, except for 12 dogs which were removed from the dataset (Supplementary Figure S5). Purebred dogs with WGS in Dog10K were then compared to breeds with known prevalence for HD, ED, and ACL rupture (Witsberger et al., 2008; Oberbauer et al., 2017). For OFA HD and ED, there were 230 dogs from 27 breeds (169 males, 54 females, and 7 unknown) with WGS data (Table 1) (Oberbauer et al., 2017). For hospital HD and ACL rupture, there were 279 dogs from 38 breeds (204 males, 56 females, and 19 unknown) with WGS data (Witsberger et al., 2008) (Table 1).

**TABLE 1:** Breed phenotypes for hip dysplasia, elbow dysplasia, and anterior cruciate ligament rupture.

Breed	Prevalence of OFA HD (Oberbauer et al., 2017)	Prevalence of OFA ED (Oberbauer et al., 2017)	Number of dogs, OFA WGS group	Prevalence of hospital HD (Witsberger et al., 2008)	Prevalence of hospital ACL rupture (Witsberger et al., 2008)	Number of dogs, hospital WGS group
Airedale Terrier	–	–	–	6.22	3.22	3
Alaskan Malamute	10.83	2.97	3	7.8	3.25	3
American Cocker Spaniel	–	–	–	0.87	2.36	1
American Staffordshire Terrier	24.39	16.07	2	1.84	4.05	2
Australian Cattle Dog	13.44	9.8	5	–	-	-
Australian Shepherd	5.33	3.5	2	3.06	2.14	2
Basset Hound	–	–	–	1.6	1.11	5
Beagle	–	–	–	0.62	2.41	5
Bearded Collie	5.3	1.94	8	–	–	–
Belgian Malinois	5.16	8.63	5	–	–	–
Bichon Frise	5.88	0.45	4	–	–	–
Border Collie	10.1	1.13	36	4.23	2.00	36
Boxer	10.7	0.73	1	2.12	5.24	1
Bulldog	–	–	–	4.42	5.33	1
Bullmastiff	23.97	14.2	2	–	–	–
Cavalier King Charles Spaniel	11.35	0.33	3	–	–	–
Chihuahua	–	–	–	0.23	2.04	4
Chow Chow	19.17	48.63	1	6.44	4.3	1
Collie	–	–	–	1.34	0.76	1
Dachshund	–	–	–	0.15	0.21	4
Dalmatian	–	–	–	1.36	2.72	1
Doberman Pinscher	5.67	0.82	3	1.34	1.85	3
Flat Coated Retriever	3.74	0.68	2	–	–	–
German Shepherd	18.94	17.83	16	10.26	2.24	16
German Wirehaired Pointer	8.02	2.17	1	–	-	-
Golden Retriever	18.84	9.69	10	8.51	2.8	10
Greyhound	–	–	–	0.37	0.55	5
Great Dane	11.62	3.53	1	3.89	2.77	1
Great Pyrenees	8.71	1.45	1	–	–	–
Greater Swiss Mountain Dog	15.38	9.11	6	–	–	–
Havanese	6.25	5.46	3	–	–	–
Irish Wolfhound	4.47	12.3	2	–	–	–
Labrador Retriever	11.24	9.57	10	7.37	5.79	10
Leonberger	12.72	3.25	51	–	-	–
Miniature Dachshund	–	–	–	0.17	0.21	3
Miniature Poodle	–	–	–	0.51	2.95	1
Miniature Schnauzer	–	–	–	0.2	0.63	24
Newfoundland	24.75	22.7	2	17.16	8.9	2
Nova Scotia Duck Tolling Retriever	5.81	2.51	1	–	–	-
Old English Sheepdog	17.76	3.36	1	11.1	0.97	1
Pembroke Welsh Corgi	16.56	2.92	2	–	–	–
Pomeranian	–	–	–	0.59	1.43	3
Portuguese Water Dog	10.98	1.43	3	–	–	–
Pug	–	–	–	1.53	0.75	20
Rhodesian Ridgeback	4.42	4.95	4	–	–	–
Rottweiler	20.07	38.07	3	10.53	8.29	3
Saint Bernard	–	–	–	14.7	3.57	2
Scottish Terrier	–	–	–	0.12	1.32	1
Shetland Sheepdog	4.16	2.79	3	1.83	1.34	3
Shih Tzu	–	–	–	0.73	0.57	3
Siberian Husky	–	–	–	2.01	2.12	2
Standard Poodle	10.99	2.81	16	2.79	1.87	16
Tibetan Mastiff	14.26	13.84	9	–	–	–
Vizsla	6.19	1.99	2	–	–	–
Weimaraner	7.94	1.57	2	3.39	1.77	2
Welsh Springer Spaniel	10.8	1.44	4	–	–	–
West Highland White Terrier	–	–	–	0.64	2.73	19
Yorkshire Terrier	–	–	–	0.36	2.27	59
**Total**			**230**			**279**

OFA, Orthopedic Foundation for Animals; HD, hip dysplasia; ED, elbow dysplasia; ACL, anterior cruciate ligament; WGS, whole-genome sequencing; SNP, single-nucleotide polymorphism. OFA HD, and ED, breed phenotypes were assigned from known breed prevalence (Oberbauer et al., 2017). Hospital HD and ACL rupture breed phenotypes were assigned from known breed prevalence (Witsberger et al., 2008). For the OFA SNP set there were 27 breeds, and 38 breeds for the hospital SNP set. The values that are bolded are the total values that are the most important.

The 45,165,129 variants in the raw VCF file were filtered to 33,414,513 SNPs for dogs with reported prevalence for HD, ED, and ACL rupture by removing indels and retaining biallelic SNPs with a minimum quality score of 20. After SNP filtering, 7,586,942 SNPs remained in the OFA SNP set, and 7,026,774 SNPs remained in the hospital SNP set (Table 2). No samples had a genotyping rate ≤ 90%.

**TABLE 2:** Detailed reporting of SNP filtering.

		Number of SNPs	
		OFA HD and ED	Hospital HD and ACL rupture
Unfiltered biallelic with quality ≥ 20		33,414,513	33,414,513
Removed	Genotype missingness (0.01)	7,980,405	9,311,368
	Minor allele frequency (0.05)	17,845,254	17,076,042
	Heterozygous haploid cells	1,912	329
**Final filtered for association analysis**		**7,586,942**	**7,026,774**
Fail Hardy–Weinberg threshold 1E-7		1,146,594	1,661,225
Fail Hardy–Weinberg threshold 5E-5		2,868,253	3,512,525

OFA, Orthopedic Foundation for Animals; HD, hip dysplasia; ED, elbow dysplasia; ACL, anterior cruciate ligament; SNP, single-nucleotide polymorphism. SNP filtering was performed using PLINK (Purcell et al., 2007). All samples had a genotyping rate ≥ 90%, so no dogs were removed. Hardy–Weinberg statistics for each SNP were calculated. The values that are bolded are the total values that are the most important.

### Linear mixed genome-wide association study model

**FIGURE 1:**
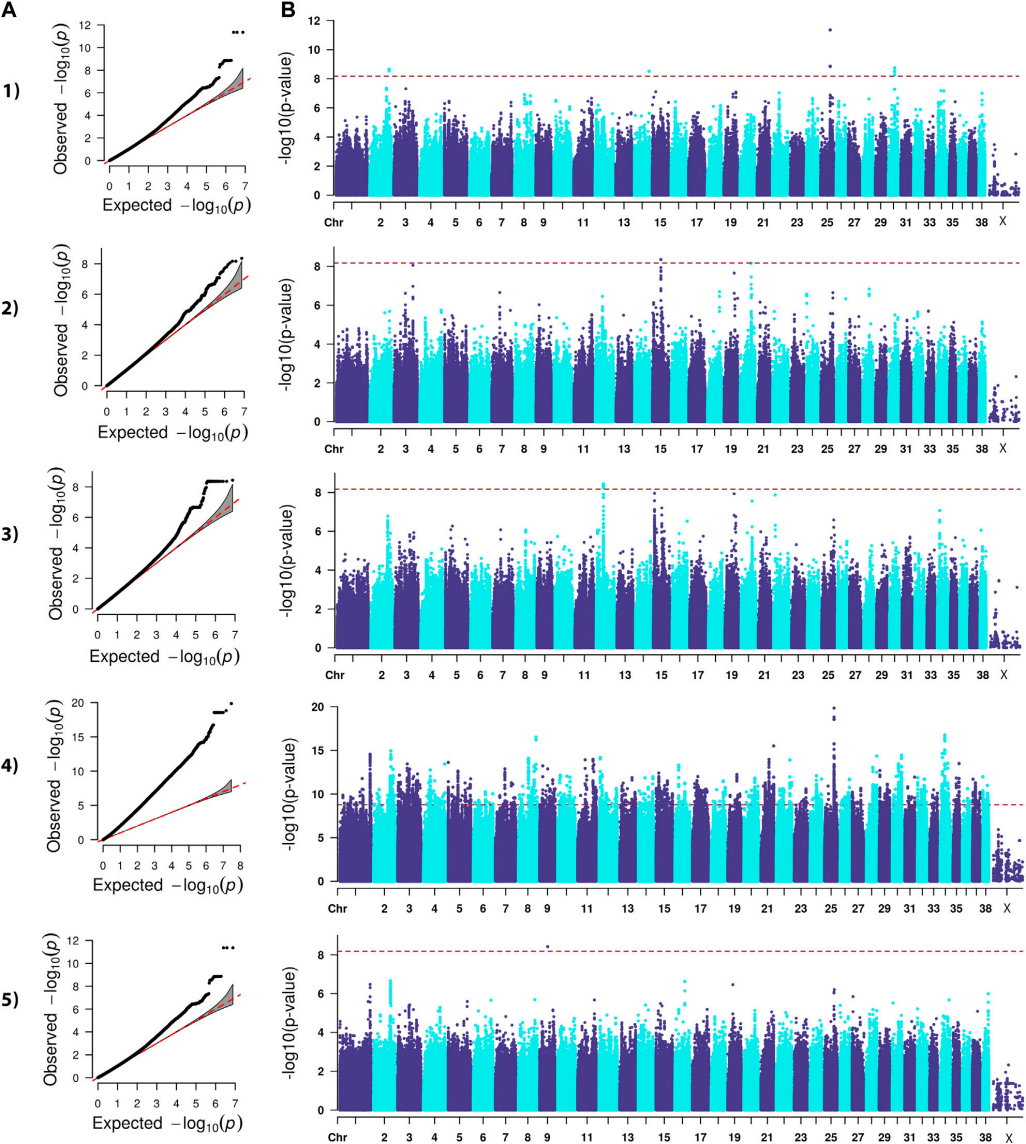
QQ **(A)** and Manhattan **(B)** plots. Comparison of four models for performing across-breed genome-wide association study using breed phenotypes for OFA hip dysplasia. 1) Linear mixed model, λ = 0.95, 16 significant single-nucleotide polymorphisms (SNPs) on chromosome 2, 14, 25, and 30 (*p* < 6.59E-9). 2) Stringent binary case-control model, λ = 1.034, 1 significant SNP on chromosome 15 (*p* < 6.68E-9). 3) Lenient binary case-control model, λ = 1.02, 21 significant SNPs on chromosome 12 (*p* < 6.64E-9). 4) Haplotype-based model, λ = 2.31, 5,687 significant SNPs with the strongest associations occurring on chromosomes 8, 21, 25, and 34 (*p* < 1.68E-9). 5) Weighted least squares model, λ = 1.54, 1 significant SNP on chromosome 9 met Bonferroni significance cutoff (*p* < 6.59E-9).

**FIGURE 2:**
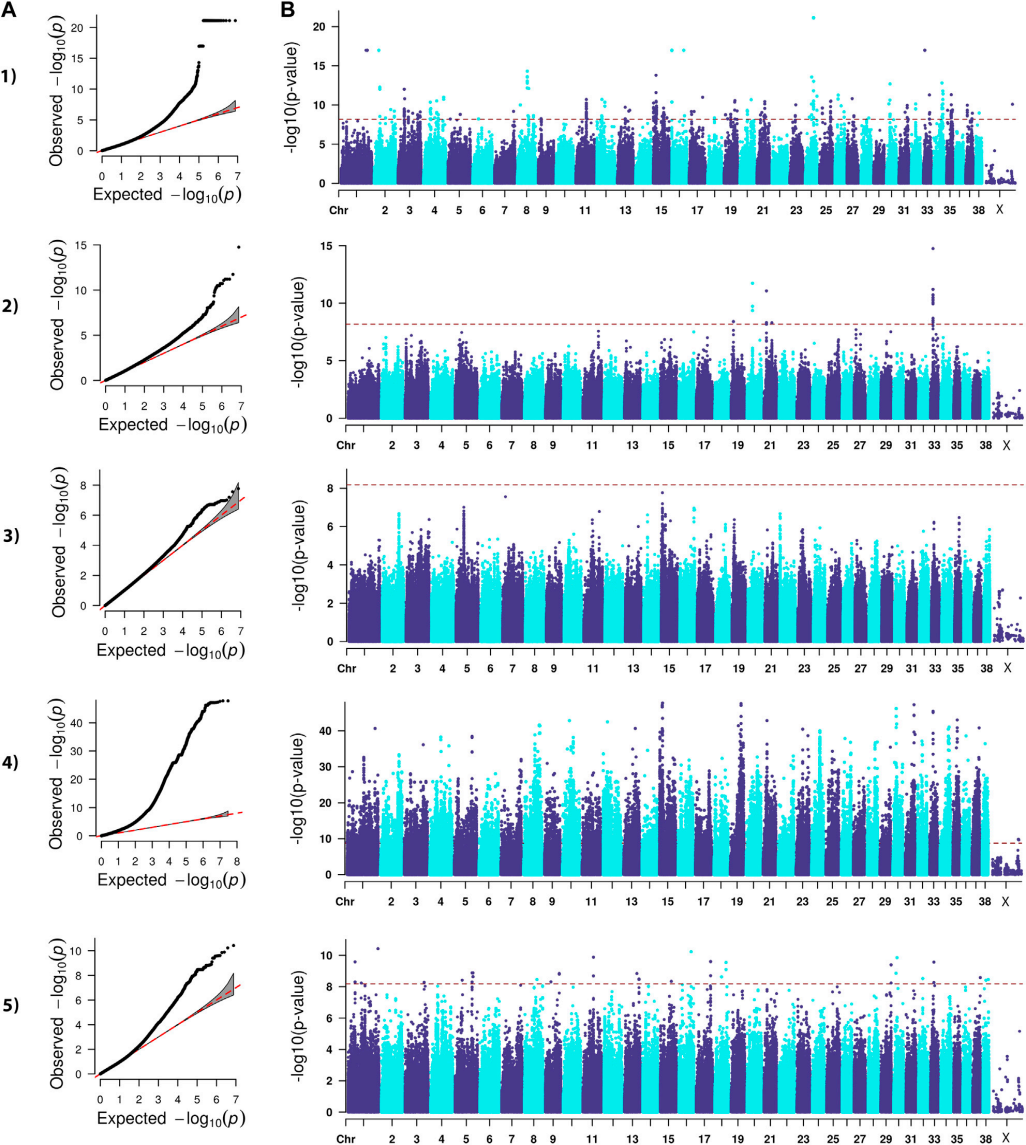
QQ **(A)** and Manhattan **(B)** plots. Comparison of four models for performing across-breed genome-wide association study using breed phenotypes for elbow dysplasia. 1) Linear mixed model, λ = 0.99, 517 significant single-nucleotide polymorphisms (SNPs) with the strongest associations on chromosome 1, 2, 8, 15, 16, 24, 30, and 33 (*p* < 6.59E-9). 2) Stringent binary case-control model, λ = 1.04, 30 significant SNPs on chromosome 19, 20, 21, 33 (*p* < 6.68E-9). 3) Lenient binary case-control model, λ = 1.01, no significant SNPs (*p* < 6.64E-9). 4) Haplotype-based model, λ = 1.84, 58,592 significant SNPs with the strongest associations on chromosomes 1, 8, 10, 12, 13, 15, 19, 21, 23, 24, 30, 31, 33, 34, 35, and 37 (*p* < 1.68E-9). 5) Weighted least squares model, λ = 0.96, 90 significant SNPs on chromosomes 1, 3, 5, 8, 9, 11, 13, 15, 16, 17, 18, 29, 30, 32, 33, 37, and 38 (*p* < 6.59E-9).

**FIGURE 3:**
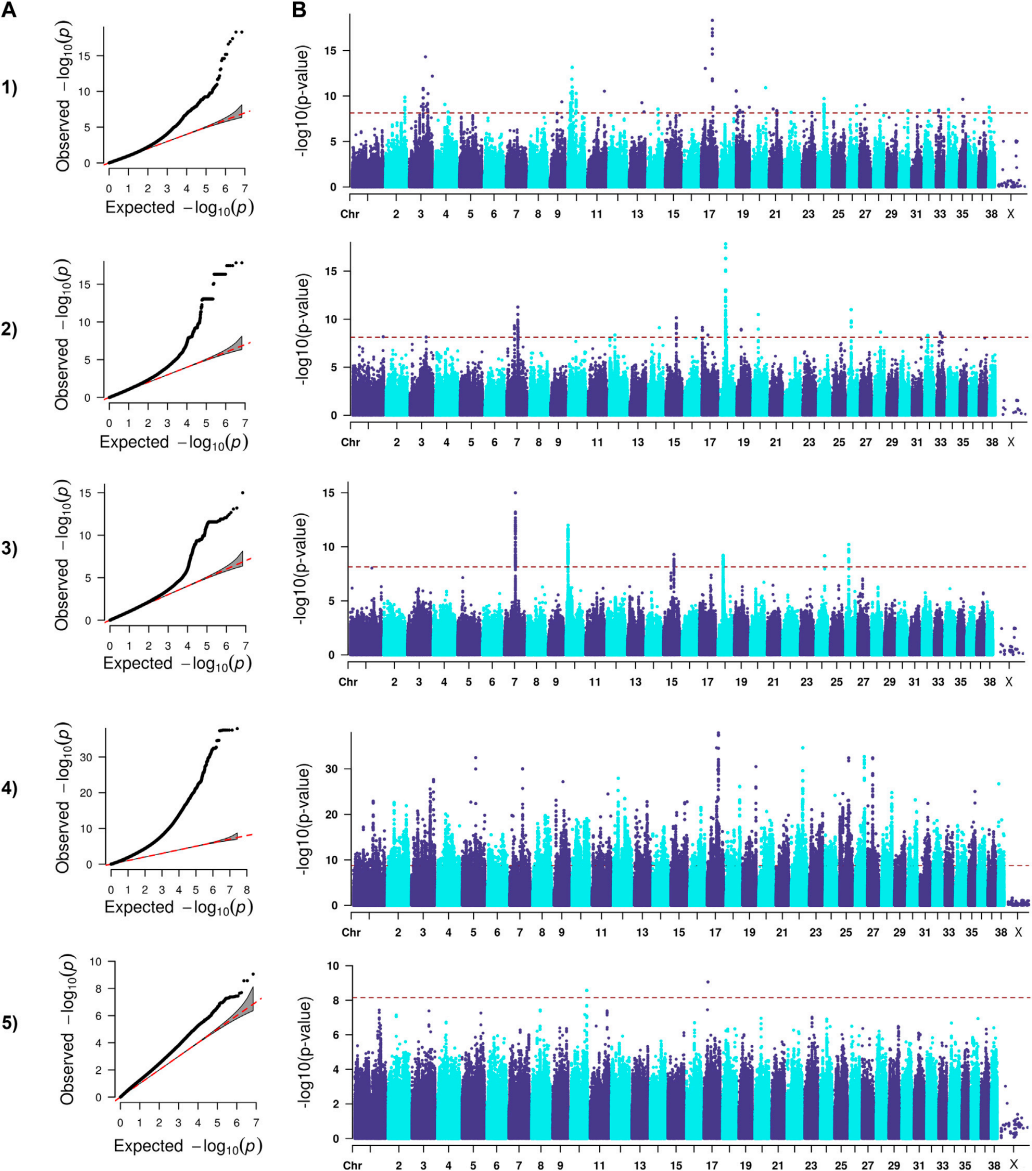
QQ **(A)** and Manhattan **(B)** plots. Comparison of four models for performing across-breed genome-wide association study using breed phenotypes for hospital hip dysplasia. 1) Linear mixed model, λ = 0.96, 195 significant single-nucleotide polymorphisms (SNPs) with the strongest associations on chromosome 3, 10, 11, 17, 19, and 20 (*p* < 7.12E-9). 2) Stringent binary case-control model, λ = 1.04, 364 significant SNPs with the strongest associations on chromosomes 7, 15, 18, 20, and 26 (*p* < 7.26.E-9). 3) Lenient binary case-control model, λ = 1.00, 376 significant SNPs on chromosomes 7, 10, 15, 18, 24, and 26 (*p* < 7.13E-9). 4) Haplotype-based model, λ = 1.97, 18,895 significant SNPs with the strongest associations on chromosomes 5, 7, 12, 17, 19, 22, 25, 26, and 27 (*p* < 1.82E-9). 5) Weighted least squares model, λ = 1.54, 3 significant SNPs on chromosomes 10 and 17 met Bonferroni significance cutoff (*p* < 7.11E-9).

**FIGURE 4:**
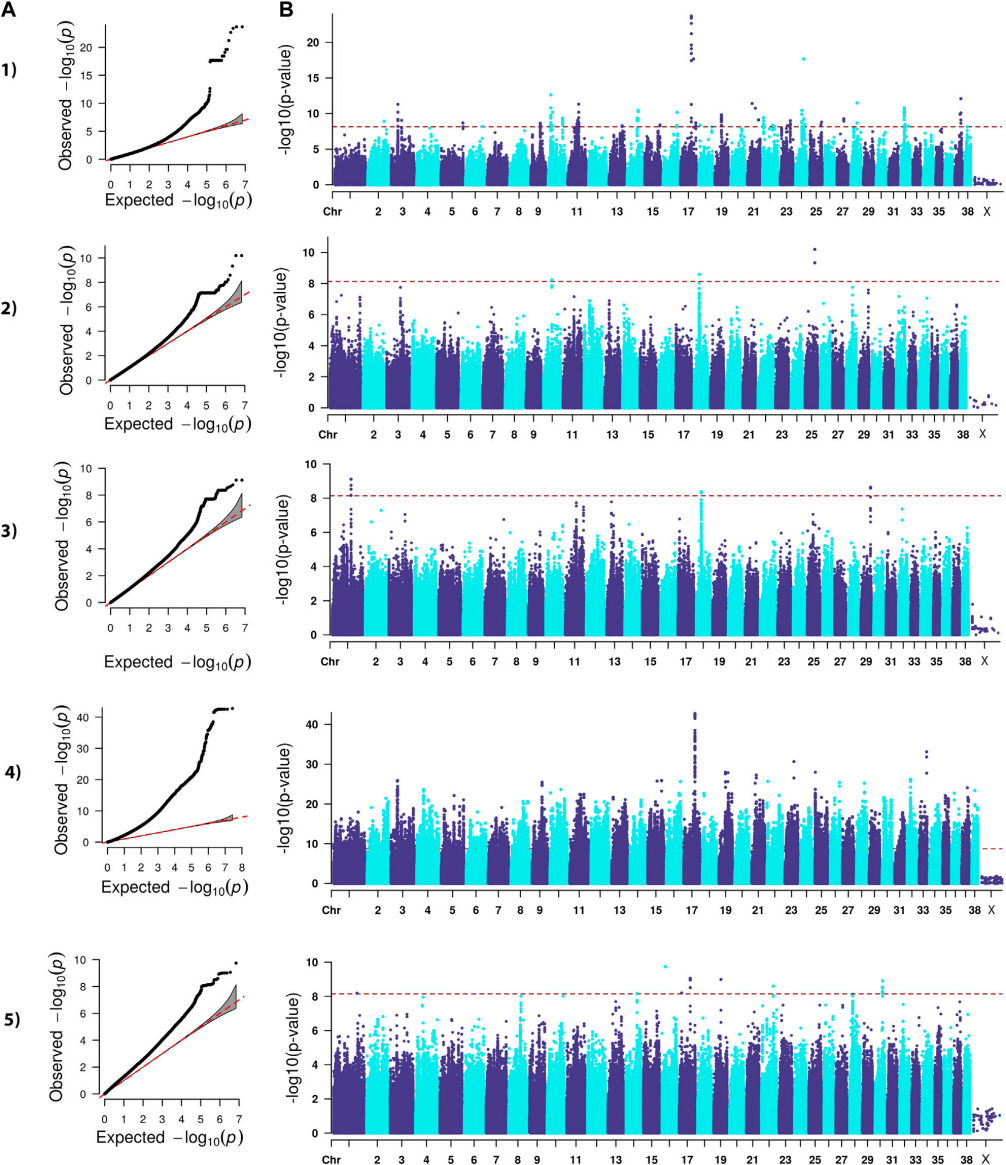
QQ **(A)** and Manhattan **(B)** plots. Comparison of four models for performing across-breed genome-wide association study using breed phenotypes anterior cruciate ligament rupture. 1) Linear mixed model, λ = 0.96, 219 significant single-nucleotide polymorphisms (SNPs) with the strongest associations on chromosomes 3, 10, 11, 14, 16, 17, 19, 21, 24, 28, 32, and 37 (*p* < 7.12E-9). 2) Stringent binary case-control model, λ = 1.05, 5 significant SNPs on chromosomes 10, 18, and 25 (*p* < 7.26E-9). 3) Lenient binary case-control model, λ = 1.03, 20 significant SNPs on chromosomes 1, 18, and 29 (*p* < 7.13E-9). 4) Haplotype-based model, λ = 2.30, 38,677 significant SNPs with the strongest associations on chromosomes 3, 9, 15, 16, 17, 19, 21, 22, 23, 25, 32, and 33 (*p* < 1.82E-9). 5) Weighted least squares model, λ = 1.58, 20 significant SNPs on chromosomes 1, 14, 16, 17, 19, 22, and 30 (*p* < 7.11E-9).

The analysis we performed was a classical generalized LMM that was corrected for population structure and sex (Figures 1–4). The number of SNPs that met genome-wide significance after Bonferroni correction was 16, 517, 195, and 219 for the OFA HD, ED, hospital HD, and ACL rupture datasets, respectively. For comparison, separate LMM FDR corrections resulted in 0, 83, 11, and 47 SNPs with significant associations for OFA HD, ED, hospital HD, and ACL rupture (Supplementary Figure S6). The SNP effect estimates are reported in Supplementary Data S5.

Lambda ranged from 0.95 for OFA HD to 0.99 for ED (Figures 1–4). The 16 significant OFA HD SNPs were located on chromosomes 2, 14, 25, and 30 (Table 3). The strongest associations for ED were located on chromosomes 1, 2, 8, 15, 16, 24, 30, and 33 (Table 4). Hospital HD SNPs had strong associations with chromosomes 3, 10, 11, 17, 19, and 20 (Table 5), and ACL rupture SNPs had strong associations on chromosomes 3, 10, 11, 14, 16, 17, 19, 21, 24, 28, 32, and 37 (Table 6). IL26 and IL22 were in a chromosome 10 region located at 10,418,984–10,524,282 bp. CSMD1 was in a region on chromosome 16 and *TNS1* was in a region on chromosome 37. The strongest association for hospital HD located on chromosome 17 at 44,162,612–44,172,578 bp overlapped with the strongest association for ACL rupture. Similarly, two significant regions on chromosome 24 at 25,363,325–25,384,175 bp and 32,828,822–32,908,498 bp were significant for both ED and ACL rupture and contained *TGIF2* and *LDHA* respectively. The chromosome 15 region located at 10,683,929–10,712,309 bp that associated with ED contained *FAF1*, and a significant hospital HD association on chromosome 3 located at 91,241,141 bp contained *LCORL*.

**TABLE 3:** Top candidate loci associated with OFA hip dysplasia in across-breed GWAS using breed phenotypes and four different association models.

Chromosome	Position/region	Number of SNPs in the region	Lowest *P* value in the region	Met Bonferroni significance	Model	Gene
2	76,620,817–76,620,931	2	2.81E-09	Y	LMM	
14	57,528,002	1	3.00E-09	Y	LMM	
25	37,396,525–37,407,286	9	4.40E-12	Y	LMM	*ENSCAFG00000043669, ENSCAFG00000021935*
30	24,560,520–24,562,383	4	1.79E-09	Y	LMM	
15	32,889,046	1	4.40E-09	Y	Stringent	
					Case-control	
12	28,194,827–28,199,583	21	3.62E-09	Y	Lenient	*ENSCAFG00000002495, ENSCAFG00000025809*
					Case-control	
8	69,277,732–69,295,716	5	2.97E-17	Y	Haplotype-based	*MIR487A, MIR544A, MIR496, ENSCAFG00000020483, ENSCAFG00000020486, ENSCAFG00000025637*
21	49,929,035–49,954,936	59	3.14E-16	Y	Haplotype-based	
25	37,223,222–46,375,929^a^ ^,HD−VET^	204	1.42E-20	Y	Haplotype-based	***COL4A3*** *,* ***COL4A4*** *,* ***FBX025,*** *ANF596, AGFG1, ARL4C, ARMC9, ATG16L1, B3GNT7, C2orf57, C2orf72, C2orf82, C8orf42, CAB39, CCDC195, CCL20, CHRND, CHRNG, COPS7B, CUL3, DGKD, DIS3L2, DNER, DOCK10, ECEL1, EFHD1, FAM124B, FB036, GIGYF2, GPR55, HEATR7B1, HJURP, HTR2B, INPP5D, IPP5D, IRS1, ITM2C, KCNJ13, NCL, NEU2, NGEF, NMUR1, NPPC, NYAP2, PDE6D, PID1, PRSSS6, PSMD1, PTMA, Q28273_CANFA, Q29468_CANFA, RHBDD1, SAG, SH3BP4, SLC16A14, SLC19A3, SNORA70, SNORC, SP100, SP11, SP110, SP140, SPATA3, SPHKAP, SPP2, TDRP, TEX, TRIP12, TRPM8, UGT1A6, WDR69, ZNF596, CFRNASEQ_PROT_00093759, ALPI, ALPG, ENSCAFG00000010555, ENSCAFG00000011227, ENSCAFG00000011328, ENSCAFG00000011833, ENSCAFG00000011889, ENSCAFG00000029276, ENSCAFG00000029715, ENSCAFG00000030279, ENSCAFG00000030283, ENSCAFG00000031066, ENSCAFG00000031071, ENSCAFG00000031160, ENSCAFG00000031445, ENSCAFG00000031683, ENSCAFG00000031923, ENSCAFG00000032308*
34	17,535,930–19,185,794	78	1.83E-17	Y	Haplotype-based	***IGF2BP2*** *, MAGEF1, VPS8, C3orf70, EHHADH, MAP3K13, TMEM41A, LIPH, SENP2, TRA2B, ETV5, DGKG, TBCCD1, DNAJB11, RPL27, ENSCAFG00000032221*
1	118,640,052–118,640,497	2	3.32E-07	N	Weighted least squares	*TDRD12*
2	66,609,105–66,649,551	18	2.16E-07	N	Weighted least squares	*SDC3, PUM1, SIAH1*
9	31,387,114^a^	1	3.75E-09	Y	Weighted least squares	*ENSCAFG00000043496, ENSCAFG00000047685*
16	39,179,963–39,183,830^a^	4	2.30E-07	N	Weighted least squares	*ENSCAFG00000034585*
19	20,295,187	1	3.461E-07	N	Weighted least squares	
25	37,373,186–37,373,537	2	8.31E-07	N	Weighted least squares	*LZTS1*
25	38,796,893	1	6.17E-07	N	Weighted least squares	*DOCK10*

SNP, single-nucleotide polymorphism; LMM, linear mixed model. Base pair locations are reported in the CanFam3.1 assembly. Phenotypes for hip dysplasia (HD) were assigned from prevalence data from the Orthopaedic Foundation for Animals (OFA) (Oberbauer et al., 2017). Binary case-control GWAS used a stringent and a lenient scenario for breed assignment as a case or a control. Haplotype GWAS used a fixed window of 4 SNPs.

^a^ Regions that overlap with previously reported HD loci (Todhunter et al., 2005; Lavrijsen et al., 2014; Sánchez-Molano et al., 2014; Mikkola et al., 2019).

^HD−VET^Regions that overlap with hospital HD candidate loci. Genes in bold type are biological disease candidates based on current knowledge.

**TABLE 4:** Top candidate loci associated with elbow dysplasia in across-breed GWAS using breed phenotypes and four different association models.

Chromosome	Position/region	Number of SNPs in the region	Lowest *P* value in the region	Met Bonferroni significance	Model	Gene
1	99,449,521–104,345,205	5	1.05E-17	Y	LMM	*AC012313.1, ZSCAN1, ZSCAN18, ZSCAN22, ZSCAN51, A1BG, AURKC, DUXA, PEG3, OR13H1, GALP, TMSB15B, NLRP5, NLRP8, NLRP9, NLRP13, PIZ1, EPN1, U2AF2, ANF580, FIZ1, SBK2, SSC5D, SHISA7, TM190, COX6B2, SUV420H2, TMEM150B, ILI1, UBE2S, RPL28, BRSK1, HSPBP1, PPP6R1, PTPRH, SYT5, PPP1R12C, EPS8L1, GP6, NCR1, CDC42EP5, TTYH1, TNNT1, LENG1, LENG8, TSEN24, CNOT3, PRPF31, TFPT, RDH13, RSP5, RPS9, CACNG8, VSTM1, RNF183, ZNF132, ZNF134, ZNF262, ZNF264, ZFP28, ZNF444, ZNF460, ZNF470, ZNF524, ZNF547, ZNF548, ZNF549, ZNF550, ZNF583, ZNF586, ZNF606, ZNF628, ZNF648, ZNF667, ZNF671, ZNF772, ZNF787, ZNF8, ZNF835, ZNF837, ENSCAFG00000002360, ENSCAFG00000002419, ENSCAFG00000002441, ENSCAFG00000002453, ENSCAFG00000002524, ENSCAFG00000002638, ENSCAFG00000002660, ENSCAFG00000002723, ENSCAFG00000023244, ENSCAFG00000023300, ENSCAFG00000014217,ENSCAFG00000024642, ENSCAFG00000002736, ENSCAFG00000028453, ENSCAFG00000028494, ENSCAFG00000028550, ENSCAFG00000028855*
2	18,386,156–18,388,527	2	1.05E-17	Y	LMM	*POTEA*
8	39,657,551–39,670,445	11	4.87E-15	Y	LMM	*ENSCAFG00000048925*
15	10,683,929–10,712,309	2	1.61E-14	Y	LMM	***FAF1*** *, DMRTA2, ENSCAFG00000041341, ENSCAFG00000045079*
16	99,257–132,953	19	1.05E-17	Y	LMM	*HUS1, PKD1L1*
16	170,017–176,415	2	1.05E-17	Y	LMM	*PKD1L1*
16	47,296,617–50,456,864	6	1.05E-17	Y	LMM	*DCTD, ODZ3, TENM3, ENSCAFG00000029296, ENSCAFG00000037207, ENSCAFG00000042580, ENSCAFG00000044307, ENSCAFG00000044369, ENSCAFG00000046295, ENSCAFG00000046706, ENSCAFG00000046838, ENSCAFG00000046895, ENSCAFG00000048385, ENSCAFG00000049167*
24	25,363,325–25,384,175^ACLR^	8	2.71E-14	Y	LMM	***TGIF2***
24	32,708,150–33,127,877^ACLR^	58	7.59E-22	Y	LMM	*PIGT, SPINLW1, CE10, DNTTIP1, WFDC2, WFDC3, WFDC6, WFDC8, WFDC9, WFDC10A, WFDC10B, WFDC11, WFD13, SPINT4, AL050348.1, ENSCAFG00000009716, ENSCAFG00000009724, ENSCAFG00000029223, ENSCAFG00000030724*
30	13,958,442	1	2.09E-13	Y	LMM	*SEMA6D*
33	5,305,089–5,305,273	8	1.05E-17	Y	LMM	
19	9,012,897–9,019,048	3	3.97E-09	Y	Stringent	
					Case-control	
20	21,930,609–21,941,275	3	1.83E-12	Y	Stringent	
					Case-control	
21	9,874,577–9,881,322	4	8.46E-12	Y	Stringent	
					Case-control	
21	31,476,485	1	5.06E-09	Y	Stringent	
					Case-control	
33	9,739,834–9,796,151	19	1.78E-15	Y	Stringent	*ENSCAFG00000009706*
					Case-control	
1	110,678,772–110,678,890	2	2.15E-41	Y	Haplotype-based	*ZNF180*
8	62,931,932–62,934,171	7	2.20E-42	Y	Haplotype-based	
8	64,808,440–64,843,448	13	4.02E-42	Y	Haplotype-based	*C14orf132*
10	28,482,503	1	1.40E-43	Y	Haplotype-based	*RBFOX2*
12	17,341,130–17,343,114	3	2.98E-43	Y	Haplotype-based	*CENPQ*
13	46,119,312–46,119,503	2	2.15E-41	Y	Haplotype-based	*LNX1*
15	10,537,302–10,541,074	3	6.81E-48	Y	Haplotype-based	***FAF1***
15	11,705,371–11,712,008	7	3.28E-44	Y	Haplotype-based	*BEND5*
15	11,780,177–11,781,722	3	1.78E-48	Y	Haplotype-based	*BEND5*
19	31,892,082	1	3.77E-42	Y	Haplotype-based	*CCDC93*
19	42,672,420–42,717,082	35	2.53E-48	Y	Haplotype-based	*LRP1B*
21	15,286,410	1	1.40E-43	Y	Haplotype-based	*DLG2*
23	26,273,374–26,375,208	35	3.42E-41	Y	Haplotype-based	*RFTN1*
24	33,101,055–33,127,398	31	9.93E-41	Y	Haplotype-based	*WFDC3, DNTTP1*
30	13,527,625–13,531,258	9	6.26E-47	Y	Haplotype-based	*ENSCAFG00000044425*
30	15,467,378–15,782,862	21	1.20E-42	Y	Haplotype-based	*FGF7, GALK2, FAM227B, DTWD1*
31	34,801,518–34,802,216	5	5.23E-48	Y	Haplotype-based	*DSCAM*
33	13,264,100–13,268,598	3	7.43E-46	Y	Haplotype-based	
34	32,911,648–32,990,376	10	3.27E-41	Y	Haplotype-based	
34	36,353,908–36,353,924	2	9.15E-42	Y	Haplotype-based	*FNDC3B*
35	17,204,890–17,207,357	8	9.15E-44	Y	Haplotype-based	*RNF144B*
37	28,338,090–28,339,624	12	1.46E-41	Y	Haplotype-based	
1	27,440,900–27,449,404	5	2.60E-10	Y	Weighted least squares	*EYA4*
1	52,342,977	1	5.84E-09	Y	Weighted least squares	*ENSCAFG00000000770, ENSCAFG00000036893*
1	118,653,633	1	3.76E-11	Y	Weighted least squares	*TDRD12*
3	75,449,096	1	5.27E-09	Y	Weighted least squares	***UGDH*** *, ENSCAFG00000035964*
5	28,001,256	1	3.83E-09	Y	Weighted least squares	*PDGFD*
5	67,307,359–67,338,933	3	1.31E-09	Y	Weighted least squares	*ENSCAFG00000048505*
5	70,865,921–70,869,961	7	1.31E-09	Y	Weighted least squares	
8	49,742,723	1	3.49E-09	Y	Weighted least squares	*TMEM63C, ANGEL1*
9	21,842,593	1	4.85E-09	Y	Weighted least squares	*WIPF2*
9	54,961,092–54,979,083	9	1.44E-09	Y	Weighted least squares	*STXBP1, SPTAN1, ENSCAFG00000020049, ENSCAFG00000055520*
11	41,857,755–41,953,735	2	1.31E-10	Y	Weighted least squares	*IZUMO3*
13	49,045,021	1	1.44E-09	Y	Weighted least squares	*REST, IGFBP7, ENSCAFG00000002254*
13	57,506,400–57,515,748	35	3.20E-09	Y	Weighted least squares	
15	43,294,871–43,295,101	2	4.56E-09	Y	Weighted least squares	*ENSCAFG00000042681, ENSCAFG00000042272*
16	48,544,967	1	5.89E-11	Y	Weighted least squares	
17	57,061,988–57,070,341	3	2.44E-10	Y	Weighted least squares	*SEC22B*
18	26,932,991	1	2.34E-09	Y	Weighted least squares	*ENSCAFG00000047020, ENSCAFG00000044444*
18	44,751,275–44,775,897	2	2.84E-10	Y	Weighted least squares	*TNNT3*
29	41,107,270–41,108,444	4	4.02E-10	Y	Weighted least squares	*CPQ, ENSCAFG00000009482*
30	9,518,967	1	1.38E-09	Y	Weighted least squares	*SNAP23*
30	13,524,047	1	1.40E-10	Y	Weighted least squares	*ENSCAFG00000044425*
32	17,088,117	1	3.01E-09	Y	Weighted least squares	
33	13,250,803–13,263,194	3	2.70E-10	Y	Weighted least squares	
37	29,748,194	1	2.62E-09	Y	Weighted least squares	*ACSL3, SERPINE2*
38	12,143,338	1	3.86E-09	Y	Weighted least squares	*ESRRG, ENSCAFG00000010767*
38	21,315,979	1	3.50E-09	Y	Weighted least squares	*PCP4L, TOMM40L, USP21*

SNP, single-nucleotide polymorphism; LMM, linear mixed model. Base pair locations are reported in the CanFam3.1 assembly. Phenotypes for elbow dysplasia (ED) were assigned from reported prevalence data from the Orthopaedic Foundation for Animals (OFA) (Oberbauer et al., 2017). Binary case-control GWAS used a stringent and a lenient scenario for breed assignment as a case or a control. Haplotype GWAS used a fixed window of 4 SNPs.

^ACLR^Regions that overlap with candidate loci for anterior cruciate ligament rupture. Genes in bold type are biological disease candidates based on current knowledge.

**TABLE 5:** Top candidate loci associated with hospital hip dysplasia in across-breed GWAS using breed phenotypes and four different association models.

Chromosome	Position/region	Number of SNPs in the region	Lowest *P* value in the region	Met Bonferroni significance	Model	Gene
3	54,856,508–54,941,403	19	1.41E-11	Y	LMM	*TM6SF1*
3	64,006,634	1	4.85E-15	Y	LMM	*ENSCAFG00000046128*
3	73,916,752	1	5.50E-11	Y	LMM	
3	91,241,141	1	6.47E-13	Y	LMM	***LCORL***
10	11,657,322–16,184,135	13	4.61E-11	Y	LMM	*ATXNL3B, CAPS2, CNOT2, C12orf28, GLIPR1L1, KCNC2, KCNMB4, LGR5, MYRFL, OR6C35, OR6C38, PTPRB, PTPRR, RAB21, RAB3IP, TBC1D15, TSPAN8, TPH2, TRHDE, U6, ZFC3H1, ENSCAFG00000000456, ENSCAFG00000000489, ENSCAFG00000000528, ENSCAFG00000023219., ENSCAFG00000030169, ENSCAFG00000031351, ENSCAFG00000031600, ENSCAFG00000037480, ENSCAFG00000038541, ENSCAFG00000042035, ENSCAFG00000044682, ENSCAFG00000045499, ENSCAFG00000046336, ENSCAFG00000047760, ENSCAFG00000047888, ENSCAFG00000049376, ENSCAFG00000049957*
10	16,184,135	1	1.40E-12	Y	LMM	
10	17,526,189–17,616,855	10	7.16E-14	Y	LMM	*ENSCAFG00000045125, ENSCAFG00000047385*
10	33,500,317–33,510,720	34	5.17E-11	Y	LMM	*SLC41A2*
11	66,826,342	1	2.93E-11	Y	LMM	*PTBP3*
17	16,207,990	1	9.22E-14	Y	LMM	
17	44,162,612–44,172,578^ACLR^	13	4.82E-19	Y	LMM	*CTNNA2, ENSCAFG00000008115*
19	119,058	1	2.97E-11	Y	LMM	*GAB1*
19	163,327–175,657	3	2.70E-11	Y	LMM	
20	53,747,513	1	1.23E-11	Y	LMM	*GTF2F1, KHSRP*
7	30,167,956–30,840,397	5	4.76E-10	Y	Stringent	*ADCY10, DCAF6, GPR161, MPC2, MPZL1, RCSD1, SFT2D2,* ***TBX19*** *, TIPRL, U2, U3, XCL1, XCL2*
					Case-control	
7	43,702,273–43,868,543	47	5.40E-12	Y	Stringent	***SMAD2,*** *ENSCAFG00000041558*
					Case-control	
15	41,216,098–41,252,205^a^	14	7.04E-11	Y	Stringent	***IGF1***
					Case-control	
18	20,169,067–21,335,100^ACLR^	263	1.47E-18	Y	Stringent	***GNAT3*** *,* ***CD36*** *, HGF, SEMA3C, ENSCAFG00000038568*
					Case-control	
20	21,975,209–21,975,837	2	3.19E-11	Y	Stringent	*ENSCAFG00000043758*
					Case-control	
26	12,553,216–12,748,621	6	9.70E-12	Y	Stringent	*ENSCAFG00000044312, ENSCAFG00000047621, ENSCAFG00000048883*
					Case-control	
7	43,702,273–43,887,371	86	9.95E-16	Y	Lenient	***SMAD2,*** *ENSCAFG00000041558*
					Case-control	
10	7,955,660–8,403,568	244	1.01E-12	Y	Lenient	***MSRB3*** *,* ***HMGA2*** *, U6, ENSCAFG00000047717, ENSCAFG00000044689*
					Case-control	
15	41,216,098–41,252,205^a^	10	5.15E-10	Y	Lenient	***IGF1***
					Case-control	
18	20,390,458–21,335,100^ACLR^	27	6.20E-10	Y	Lenient	*SEMA3C, HGF, ENSCAFG00000038568*
					Case-control	
24	34,914,786	1	6.85E-10	Y	Lenient	
					Case-control	
26	12,553,216–12,748,621	8	1.72E-10	Y	Lenient	*ENSCAFG00000044312, ENSCAFG00000047621, ENSCAFG00000048883*
					Case-control	
5	52,642,429–52,642,493	2	3.40E-33	Y	Haplotype-based	*C8A*
7	49,528,690–51,225,042	89	9.39E-31	Y	Haplotype-based	*SNORD22, ENSCAFG00000033193, ENSCAFG00000034599, ENSCAFG00000036953, ENSCAFG00000038153, ENSCAFG00000044775, ENSCAFG00000044870, ENSCAFG00000045965, ENSCAFG00000047144, ENSCAFG00000048297*
12	19,131,180–19,137,368	9	1.16E-28	Y	Haplotype-based	
17	35,125,440–37,641,224	215	2.30E-35	Y	Haplotype-based	*ACOXL, ANAPC1, BCL2L11, BUB1, CD24, CKAP2L, CHCHD5, FBLN7, IGKV3-11, IGKV3-15, IGKV3D-20, IGKV3D-7,* ***IL1A*** *,* ***IL1B*** *, IL1F10, IL1RA, IL36B, IL36RN, IL37, MERTK, NPHP1, NT5DC4 PAX8, POLR1B, PSD4, SLC20A1, TMEM87B, TPC3, TTL, ZC3H6, ZC3H8, ENSCAFG00000001564, ENSCAFG00000007366, ENSCAFG00000015557, ENSCAFG00000023277, ENSCAFG00000029356, ENSCAFG00000029479, ENSCAFG00000031118, ENSCAFG00000031795, ENSCAFG00000031853, ENSCAFG00000032762*
17	43,708,788–45,768,418^a^ ^,ACLR^	136	1.21E-38	Y	Haplotype-based	*CTNNA2, CTSV, LRRTM1,* ***REG3A*** *, ENSCAFG00000008109, ENSCAFG00000008115, ENSCAFG00000031191, ENSCAFG0000004095*
19	52,085,604–52,278,356	7	3.12E-31	Y	Haplotype-based	*NXF2, NXF2B, ENSCAFG00000046301*
22	42,751,590–48,024,084^ACLR^	205	2.30E-35	Y	Haplotype-based	*ABCC4, CLDN10, DCT, DNAJC3, DZIP1, GPC5, GPC6, GPR180, HS6ST3, MBNL2, OXGR1, RAP2A, TGDS, UGGT2, ENSCAFG00000005471, ENSCAFG00000025482, ENSCAFG00000028798, ENSCAFG00000036423, ENSCAFG00000036528, ENSCAFG00000043028, ENSCAFG00000043306, ENSCAFG00000044606, ENSCAFG00000047495, ENSCAFG00000049489*
25	37,334,404–41,844,408^a^ ^,HD-OFA^	60	5.10E-21	Y	Haplotype-based	*FBXO25, C8orf42, FAM124B, CUL3, DOCK10, NYAP2, IRS1, RHBDD1, AGFG1, TM4SF20, MFF, CCDC195, SLC19A3, CCL20, WDR69, SPHKAP, DNER, PID1, ENSCAFG00000031160, Q28273_CANFA, CFRNASEQ_PROT_00093757, CFRNASEQ_PROT_00093826*
26	35,741,920–35,742,044	3	3.52E-21	Y	Haplotype-based	*PRKG1*
26	35,761,176–35,761,584	3	2.02E-33	Y	Haplotype-based	*PRKG1*
27	20,410,196–20,417,696	11	3.83E-33	Y	Haplotype-based	*ARNTL2*
27	20,510,657–20,511,216	4	3.83E-33	Y	Haplotype-based	
1	96,778,387–96,778,618	2	3.68E-08	N	Weighted least squares	
2	32,340,678–32,343,102	2	7.22E-08	N	Weighted least squares	*NRG2*
3	67,176,005	1	4.16E-08	N	Weighted least squares	
5	74,386,893	1	5.49E-08	N	Weighted least squares	
8	34,351,846–34,394,055	7	3.68E-08	N	Weighted least squares	*DAAM1*
10	64,779,596–64,780,180^a^	2	2.69E-09	Y	Weighted least squares	***SPRED2***
11	66,711,952–67,212,803	11	4.12E-08	N	Weighted least squares	***COL27A1,*** *PTBP3, HSDL2, KIAA 1958, INIP, ZNF618, TMEM268, AMBP, KIF12, WHRN, AKNA, SNX30, ENSCAFG00000032333, ENSCAFG00000003039, ENSCAFT00000004889, ENSCAFG00000003331, CFRNASEQ_PROT_00019705*
17	14,236,771	1	3.56E-08	N	Weighted least squares	*ENSCAFG00000042223*
17	16,207,990	1	8.71E-10	Y	Weighted least squares	
23	28,861,948	1	9.51E-08	N	Weighted least squares	*CPNE4*

SNP, single-nucleotide polymorphism; LMM, linear mixed model. Base pair locations are reported in the CanFam3.1 assembly. Phenotypes for hip dysplasia (HD) were assigned from reported hospital prevalence data (Witsberger et al., 2008). Binary case-control GWAS used a stringent and a lenient scenario for breed assignment as a case or a control. Haplotype GWAS used a fixed window of 4 SNPs.

^a^ Regions that overlap with previously reported HD loci (Todhunter et al., 2005; Zhou et al., 2010; Sánchez-Molano et al., 2014; Fealey et al., 2017).

^HD−OFA^Regions that overlap with OFA HD candidate loci.

^ACLR^Regions that overlap with anterior cruciate ligament rupture candidate loci. Genes in bold type are biological disease candidates based on current knowledge.

**TABLE 6:** Top candidate loci associated with anterior cruciate ligament rupture in across-breed GWAS using breed phenotypes and four different association models.

Chromosome	Position/region	Number of SNPs in the region	Lowest *P* value in the region	Met Bonferroni significance	Model	Gene
3	26,203,033–26,221,508	5	4.88E-12	Y	LMM	*RASGRF2*
10	10,418,984–10,524,282	9	2.29E-13	Y	LMM	***IL26***, ***IL22***, *MDM1*
10	17,861,930–17,865,318	4	6.22E-11	Y	LMM	
11	43,767,715–43,801,952	14	4.70E-12	Y	LMM	*ENSCAFG00000007543*
14	43,752,979–43,754,827	4	3.48E-11	Y	LMM	*ADCYAP1R1*
16	55,863,899	1	6.89E-11	Y	LMM	***CSMD1***
17	44,162,612–44,182,124^HD−VET^	17	2.14E-24	Y	LMM	*CTNNA2, ENSCAFG00000008115*
17	53,020,699	1	2.13E-18	Y	LMM	
19	25,995,554–26,035,873	19	1.47E-10	Y	LMM	*CNTP5, CNTNAP5*
21	18,414,027	1	3.87E-12	Y	LMM	
21	31,381,133	1	1.78E-11	Y	LMM	
24	25,363,325–25,411,513^ED^	12	3.49E-11	Y	LMM	***TGIF2*** *, NDRG3, RAB5IF, SLA2*
24	32,828,822–32,908,498^ED^	42	2.13E-18	Y	LMM	*CE10,* ***LDHA*** *, ENSCAFG00000009716*
28	25,261,122–25,400,108	2	3.18E-12	Y	LMM	*ABLIM1*
32	13,944,414–13,999,383	8	1.60E-11	Y	LMM	*FAM190A*
37	21,377,054–21,377,372	3	1.27E-10	Y	LMM	*SPAG16, ENSCAFG00000032634*
37	24,674,806–24,675,939	4	8.08E-13	Y	LMM	***TNS1***
10	33,292,773	1	5.84E-09	Y	Stringent	*WASHC4*
					Case-control	
18	21,314,959^HD−VET^	1	2.57E-09	Y	Stringent	
					Case-control	
25	38,714,863–38,714,897	3	6.36E-11	Y	Stringent	*NYAP2*
					Case-control	
1	74,908,086–74,980,615	6	7.74E-10	Y	Lenient	*ENSCAFG00000047840*
					Case-control	
18	20,225,143–20,303,181^HD−VET^	12	4.27E-09	Y	Lenient	***GNAT3, CD36***
					Case-control	
29	38,293,114–38,293,125	2	2.29E-09	Y	Lenient	*ENSCAFG00000045633*
					Case-control	
3	26,239,450–26,240,409	5	1.23E-26	Y	Haplotype-based	RASGRF2
9	39,215,124–39,215,828	3	3.50E-26	Y	Haplotype-based	*ASIC2*
15	54,466,277–54,472,528	10	1.21E-26	Y	Haplotype-based	*GLRB*
16	55,863,606–55,864,050	5	2.44E-26	Y	Haplotype-based	***CSMD1***
17	44,158,860–44,173,015^HD−VET^	68	1.76E-43	Y	Haplotype-based	*CTNNA2, ENSCAFG00000008115*
19	26,058,067–26,076,374	9	1.05E-28	Y	Haplotype-based	*CNTP5, CNTNAP5*
19	35,483,999–36,323,546	15	1.40E-28	Y	Haplotype-based	*GPR39, LYPD1, NCKAP5, MSMO1*
21	17,631,572–17,632,675	2	5.54E-28	Y	Haplotype-based	
22	3,272,502–3,272,780	2	2.31E-26	Y	Haplotype-based	*ITM2B*
23	35,372,562–35,372,596	2	2.25E-31	Y	Haplotype-based	*ENSCAFG00000049624*
25	1,690,842–1,710,200	7	1.10E-28	Y	Haplotype-based	*LHFP*
32	13,944,097–13,945,336	7	6.00E-27	Y	Haplotype-based	*FAM190A*
33	29,413,308–29,444,240	4	7.75E-34	Y	Haplotype-based	*PCYT1A*, *TM4SF19*
1	93,636,376	1	6.50E-09	Y	Weighted least squares	*PDCD1LG2, ENSCAFG00000002121*
14	43,753,764–43,753,925	2	6.85E-09	Y	Weighted least squares	*ADCYAP1R1, PDE1C*
16	13,815,497	1	1.82E-10	Y	Weighted least squares	*PGBD2, CNOT4, ENSCAFG00000003149*
17	9,880,791	1	6.27E-09	Y	Weighted least squares	*ENSCAFG00000036378*
17	44,167,247–44,171,925^HD−VET^	7	8.76E-10	Y	Weighted least squares	*CTNNA2, ENSCAFG00000008079, ENSCAFG00000008115*
19	28,412,044	1	1.02E-09	Y	Weighted least squares	*ENSCAFG00000037290*
22	47,907,808^HD−VET^	1	2.56E-09	Y	Weighted least squares	*IPO5*
30	29,895,250–29,897,042	6	1.21E-09	Y	Weighted least squares	*SLC24A1, PTPLAD1*

SNP, single-nucleotide polymorphism; LMM, linear mixed model. Base pair locations are reported in the CanFam3.1 assembly. Phenotypes for anterior cruciate ligament (ACL) rupture were assigned from hospital prevalence data (Witsberger et al., 2008). Binary case-control GWAS used a stringent and a lenient scenario for breed assignment as a case or a control. Haplotype GWAS used a fixed window of 4 SNPs.

^HD−VET^Regions that overlap with hospital hip dysplasia candidate loci. Genes in bold type are biological disease candidates based on current knowledge.

### Binary case-control genome-wide association study model

A stringent and a less stringent scenario were used for breed assignment (Supplementary Table S2–S6; Supplementary Data S4). The stringent case-control scenario for OFA HD had 1 SNP on chromosome 15 that met genome-wide significance. With the less stringent scenario, 21 SNPs on chromosome 12 met genome-wide significance (Figure 1) (Table 3). With the stringent scenario, 30 significant ED SNPs were found on chromosomes 19, 20, 21, and 33. No significant SNPs were identified with the less stringent scenario (Figure 2) (Table 4). For hospital HD, 364 significant SNPs were found with the stringent scenario with the strongest associations located on chromosomes 7, 15, 18, 20, and 26 (Figure 3), and 376 significant SNPs on chromosomes 7, 10, 15, 18, 24, and 26 were found with the less stringent scenario. Genes within candidate regions for hospital HD included *IGF1*, *TBX19,* *GNAT3*, *CD36*, *MSRB3*, *HMGA2*, and *SMAD2* (Table 5)*.* For ACL rupture, 5 significant SNPs were found with the stringent scenario on chromosomes 10, 18, and 25, while the lenient scenario identified 20 SNPs on chromosomes 1, 18, and 29 (Figure 4). Two ACL rupture candidate loci on chromosome 18 located at 20,225,143–20,303,181bp and 21,314,959bp overlapped with candidate loci for hospital HD. The SNP effect estimates are reported in Supplementary Data S5.

### Haplotype-based genome-wide association study model

Scree plots (Supplementary Figure S7) were used to determine the optimum number of eigenvectors for the correction of population structure. For the OFA HD and ED SNPs, the first six eigenvectors were used as covariates, and the first eight eigenvectors were used for the hospital HD and ACL rupture SNPs. When the haplotype-based analysis was performed with a fixed 4 SNP window, 7,586,822 and 7,026,651 sliding windows were analyzed for the OFA and hospital datasets, respectively. Bonferroni genome-wide significance was set at (cutoff = -log10 (0.05/number of haplotypes) = 8.77 (OFA dataset) and 8.74 (veterinary hospital dataset). Lambda values ranged from 1.84 (ED) to 2.31 (OFA HD) (Figures 1–4). For OFA HD there were 5,687 significant SNPs, with the strongest associations located on chromosomes 8, 21, 25, and 34 (Figure 1). The locus on chromosome 34 contained *IGF2BP2,* a gene known to influence body size. For ED, there were 58,592 significant SNPs, with the strongest associations located on chromosomes 1, 8, 10, 12, 13, 15, 19, 21, 23, 24, 30, 31, 33, 34, 35, and 37 (Figure 2) (Table 4). The chromosome 15 loci 10,537,302–10,541,074bp contained the gene *FAF1*. For hospital HD there were 18,895 significant SNPs with the strongest associations on chromosomes 5, 7, 12, 17, 19, 22, 25, 26, and 27 (Figure 3). The chromosome 17 locus, which contained *REG3A,* overlapped with an ACL rupture locus from the LMM, haplotype, and weighted least square models. The chromosome 25 locus also overlapped with an OFA HD locus determined by the haplotype model. The chromosome 17 locus 35,125,440–37,641,224 bp contained the IL1A and IL1B genes. For ACL rupture, there were 38,677 significant SNPs, with the strongest associations located on chromosomes 3, 9, 15, 16, 17, 19, 21, 22, 23, 25, 32, and 33. The chromosome 17 locus at 44,158,860–44,173,015bp overlapped with the hospital HD candidate loci. Separate haplotype-based analysis with FDR correction still had evident genomic inflation (Supplementary Figure S8). After FDR correction, 36 and 23,598 SNPs met the same Bonferroni significance thresholds for OFA HD and ED analysis. The hospital HD and ACL rupture analysis had 4,294 and 10,403 SNPs meet Bonferroni significance thresholds after FDR correction. The SNP effect estimates are reported in Supplementary Data S5.

### Weighted least squares genome-wide association study model

The final model performed for comparison was a novel weighted least squares approach. This association analysis calculated the average allelic frequency for each SNP within a breed and then weighted it according to the number of individuals (repeats) within a breed in the dataset. This model was developed to address the challenges of unbalanced breed representation in the repository data rather than excluding individuals to balance the number of dogs per breed. Genome-wide significance was set at Bonferroni correction of *p* < 6.59E-9 for the OFA HD and ED datasets and *p* < 7.11E-9 for the hospital HD and ACL rupture datasets.

Lambda values ranged from 0.96 (OFA ED) to 1.58 (ACL rupture) (Figures 1–4). 1 SNP on chromosome 9 met genome-wide significance when the OFA HD dataset was analyzed (Figure 1). This chromosome 9 SNP is an established HD locus (Table 3) (Mikkola et al., 2019). One locus on chromosome 16 that was approaching significance overlapped with an established HD locus (Table 3) (Todhunter et al., 2005). Another region approaching genome-wide significance on chromosome 25, at 37,373,186–37,373,537bp, was reported as significant with the LMM and haplotype-based GWAS. The weighted least squares analysis of the ED data resulted in 90 SNPs located on chromosomes 1, 3, 5, 8, 9, 11, 13, 15, 16, 17, 18, 29, 30, 32, 33, 37, and 38 that met genome-wide significance (Figure 2) (Table 4). The chromosome 3 locus contained the gene *UGDH.* The chromosome 16 locus was also reported as significant with the LMM GWAS. 2 SNPs on chromosome 10 and 1 SNP on chromosome 17 met genome-wide significance for the hospital HD data (Figure 3). The region on chromosome 10 (Table 5) is a known HD associated locus and contained *SPRED2* (Todhunter et al., 2005). Of the SNPs that were approaching genome-wide significance, a chromosome 11 region contained *COL27A1* and was reported as significant with the LMM GWAS. 20 SNPs located on chromosomes 1, 14, 16, 17, 19, 22, and 30, met genome-wide significance for the weighted least squares analysis of the ACL rupture dataset (Table 6). The significant locus on chromosome 14 was also reported as significant with the LMM GWAS. The chromosome 17 locus at 44,167,247–44,171,925bp was also reported as significant with the LMM and haplotype GWAS, and this region also overlapped with a hospital HD candidate locus. The SNP effect estimates are reported in Supplementary Data S5.

## Discussion

To investigate large-effect disease-associated variants for three major canine orthopedic diseases, we performed association analysis with LMM, binary case-control, haplotype-based, and weighted least squares methods with breed phenotypes. We selected the diseases to study, in part, because there is existing genetic discovery literature, particularly for HD. This body of work allowed us to compare previously reported candidate variants with the loci and genes identified in the present study.

In total, we identified six HD candidate loci on chromosomes 9, 10, 15, 16, 17, and 25 that have been previously reported in the literature. A chromosome 25 region containing the *COL4A3* and *COL4A4* genes was associated with Norberg Angle (NA), a measurement of hip joint laxity, in a population of British Labrador Retrievers (Sánchez-Molano et al., 2014), and had some overlap with another chromosome 25 region with suggestive association of HD in Dutch Labrador Retrievers (Lavrijsen et al., 2014). Portions of these regions were identified with OFA HD LMM analysis, both OFA and hospital HD haplotype analyses, and approached significance with the OFA HD weighted least squares analysis. The F-box protein 25 (*FBX025)* gene that controls ubiquitin-mediated proteolysis is within the chromosome 25 HD region of interest (Lykkjen et al., 2010). F-box proteins are important in cell signaling, transcription, and cell cycle signaling. *FBX025* can influence osteoblast differentiation (He et al., 2019). GWAS of nontraumatic osteonecrosis of the femoral head in humans reported an association with a *FBX025* SNP (Zhang et al., 2020). Subtle changes to hip endochondral ossification, femoral head shape, or subchondral bone and articular cartilage healing could all be mechanisms by which *FBX025* influences HD risk (Schachner and Lopez 2015).

A chromosome 9 locus identified by the weighted least squares OFA HD GWAS overlapped with a region previously associated with hip joint incongruity, femoral head positioning, and NA in German Shepherds (Mikkola et al., 2019). A locus on chromosome 16 with no annotated genes approached significance in the weighted least squares OFA HD GWAS and has been associated with NA in the offsprings of Greyhounds crossed with HD Labrador Retrievers (Todhunter et al., 2005). Similarly, a chromosome 10 region containing *SPRED2* was detected in the weighted least squares hospital HD GWAS and overlapped an HD quantitative trait locus in the same Greyhound–Labrador Retriever crosses (Todhunter et al., 2005). *SPRED2* functions in chondrocyte and bone development, with promoter activity noted in the acetabulum and femoral head (Bundschu et al., 2005).

With the hospital HD SNPs, haplotype GWAS found a chromosome 17 locus containing *REG3A* (regenerating islet derived gamma 3). This candidate locus is strongly associated with hip osteoarthritis (OA), suggesting *REG3A* may influence canine hip OA through MAPK pathway signaling (Zhou et al., 2010). *REG3A* plays a role in adhesion, cell recognition, and protection from oxidative stress-induced-apoptosis; *REG3A* may affect OA progression by regulating chondrocyte response to injury (Ortiz et al., 1998; Zhou et al., 2010).

The locus on chromosome 15 that was previously reported in the literature was found through both stringent and lenient case-control binary analysis of hospital HD data (Fealey et al., 2017). Interestingly, both stringent and lenient case-control GWAS identified a candidate locus on chromosome 15 containing *IGF1*, the growth hormone that influences body size and lifespan in dogs (Plassais et al., 2019). A previous across-breed GWAS identified *IGF1* as a candidate gene influencing pelvis size and risk of HD, as dogs with larger pelves tend to have smaller NA and increased HD risk (Fealey et al., 2017). *IGF1* was not the only domestication gene found within HD candidate loci. *IGF1, LCORL*, *SMAD2*, *HMGA2, TBX19*, *IGF2BP2,* *MSRB3, GNAT3,* and *CD36* are all genes that have been under selection during domestication (Cadieu et al., 2009; Zapata et al., 2016; Plassais et al., 2019). *LCORL, SMAD2, HMGA2, TBX19,* and *IGF2BP2* are well-documented regulators of dog size and are associated with domestication (Sutter et al., 2007; Sutter et al., 2008; Boyko et al., 2010; Hoopes et al., 2012; Hayward et al., 2016; Plassais et al., 2017; Plassais et al., 2019). These results suggest that the risk of HD is influenced by morphological trait selection during domestication. However, additional analysis should be pursued to correct for breed size to confirm the association with HD that is not confounded by breed differences in body size.

Other evidence that HD has been influenced by domestication is that *MSRB3, GNAT3,* and *CD36* are also genes responsible for diverse phenotypic and behavioral traits associated with domestication. *MSRB3* is associated with a dropped ear morphology (Vaysse et al., 2011; Webster et al., 2015). *GNAT3* and *CD36* have been associated with behavioral traits undergoing selection, including touch sensitivity, fear, and aggression (Zapata et al., 2016). Although most of these associations with domestication were detected using case-control binary GWAS, *LCORL*, and, *IGF2BP2* were highlighted by LMM and haplotype GWAS, respectively.

Haplotype GWAS identified a novel association between hospital HD and a candidate locus on chromosome 17 that contains *IL1A* (interleukin 1α) and *IL1B* (interleukin 1β). *IL1A* and *IL1B* polymorphisms have been associated with human hip OA patients (Cai et al., 2015). One proposed mechanism is that *IL1* transcription and synthesis promotes cartilage breakdown, the release of arachidonic acid, and prostanoid and eicosanoid production (Solovieva et al., 2009). The hospital HD weighted least squares analysis revealed a novel chromosome 11 region containing *COL27A1* that approached significance. Mutations in *COL27A1* are associated with human orthopedic disease, Steel syndrome, which includes bilateral congenital HD (Gonzaga-Jauregui et al., 2015).

LMM and haplotype GWAS identified an interesting association between ED and a locus on chromosome 15 containing *FAF1* (fas-associated factor 1)*,* an apoptosis enhancer that modulates osteoblast differentiation (Chu et al., 1995; Zhang et al., 2011). *FAF1* has been associated with osteochondrosis of the proximal radius and ulna and the distal humerus in pigs (Rangkasenee et al., 2013). In addition, the weighted least squares ED analysis detected a chromosome 3 region containing *UGDH*. *UGDH* is involved in the biosynthesis of glycosaminoglycans. A structural variant affecting *UGDH* has been associated with disproportional dwarfism in cats (Buckley et al., 2020; Struck et al., 2020). Interestingly, affected dwarf cats have rotational abnormalities present in their thoracic limbs resulting in moderate elbow incongruity (Struck et al., 2020).

We identified candidate loci for both HD and ACL rupture on chromosomes 17, 18, and 22. The chromosome 17 locus, containing *CTNNA2,* was the most significant haplotype GWAS association for both HD and ACL rupture. *GNAT3* and *CD36* are genes associated with behavioral domestication traits residing in the chromosome 18 locus (Zapata et al., 2016). Further investigation is warranted to explore shared variants across breeds and diseases. Two chromosome 24 loci were associated with both ED and ACL rupture and included *TGIF2,* a gene that influences transcription and cellular proliferation. Suppressed *TGIF2* expression results in decreased cellular proliferation, increased synovial cell apoptosis, and associated OA (Luo et al., 2019). Interestingly, we did not identify shared loci for HD and ED.

Several interesting OA genes were identified from the ACL rupture LMM GWAS. The chromosome 10 locus contained two interleukin genes, *IL26* and *IL22*. *IL22* has been positively correlated with synovitis in knee OA, suggesting a functional role (Deligne et al., 2015). A chromosome 16 locus containing *CSMD1* was detected by LMM and haplotype GWAS*.* A *CSMD1* SNP influences height and risk of OA in humans (Elliott et al., 2013). In another study, a *CSMD1* SNP was associated with CTX-II levels, a urinary biomarker of cartilage degradation (Ramos et al., 2014). Another ACL rupture locus on chromosome 24 contains *LDHA* (lactate dehydrogenase A), which catalyzes the final step of anaerobic glycolysis. In chondrocytes under inflammatory stress, LDHA binds NAHD and increases oxidation and free radical generation, thereby augmenting the inflammatory response and OA severity (Arra et al., 2020). Lastly, the chromosome 37 locus contained *TNS1* (tensin 1), which is essential for extracellular fibronectin, collagen matrix formation, and myofibroblast differentiation (Bernau et al., 2017). *TNS1* expression is decreased in ruptured ACL in patients with a meniscal tear compared to patients without meniscal injury (Brophy et al., 2018).

Classic LMMs perform a series of single SNP association tests and examine phenotype associations independently. LMMs are generally used to analyze balanced datasets with quantitative traits for phenotypes. Quantitative traits are traits that vary among individuals over a range of continuous distributions of phenotypes, such as height or weight. Haplotype-based models perform a similar analysis of quantitative traits, but rather than perform association analysis with individual SNPs, the association analysis is performed on a neighboring group of SNPs on a chromosome. Haplotype-based methods can increase statistical power to detect lower frequency variants by constructing groups of SNPs based on the principle that SNPs in close proximity are more likely to be inherited together. Binary case-control models are used to analyze dichotomous phenotypes. Classic LMM, haplotype-based, and binary case-control models can perform poorly when datasets are unbalanced, which can inflate type 1 error rates for rare phenotypic variants. In addition to performing poorly with unbalanced datasets, these methods are optimized for quantitative (LMM and haplotype-based) and dichotomous phenotypes (binary case-control). Breed phenotypes are ordinal categorical phenotypes. Using categorical phenotypes with models optimized to handle quantitative or dichotomous traits may result in lost information and decreased power (Lane et al., 2019).

Biologically relevant associations were identified by all GWAS methods with no evidence that one method is advantageous. No methodology performed consistently better across all datasets. LMM, haplotype-based, case-control binary, and weighted least squares models all reported biologically relevant associations. No two methods had a constant overlap of significant findings over all diseases analyzed. Currently, there is little literature documenting candidate loci for ED and ACL rupture, limiting validation findings and hindering complete performance assessment of the models. Many significant associations do not contain annotated genes. This could be due to imperfect annotation of the reference genome. However, such regions could contain quantitative trait loci that explain variation in gene expression and associated disease risk.

Several study limitations were identified. Genomic inflation with high lambda values was a consistent feature of haplotype GWAS. This could be due to the use of a fixed SNP window exacerbating allele frequency differences with the large, fixed haplotypes that exist across breeds of dogs (Lindblad-Toh et al., 2005). Although elevated lambda values can reflect disease polygenicity, in an across-breed GWAS using breed phenotypes, inflated values are more likely a consequence of population structure. Despite the application of FDR correction to the haplotype GWAS, genomic inflation was still evident. The fixed SNP window haplotype GWAS likely had the highest rate of reporting type 1 errors under the parameters tested in this study. Refinement of the haplotype GWAS approach appears warranted. Rather than fixed haplotypes, the use of dynamically defined haplotypes (Hamazaki and Iwata, 2020) could help address this problem.

Overall, the weighted least squares method was able to identify one SNP that was previously reported or detected by another model for all diseases. The weighted least squares method most often identified a moderate number of SNPs, but the Bonferroni correction appeared too conservative as even loci that had been previously reported did not meet genome-wide significance, such as the chromosome 16 locus identified from OFA HD SNPs. The advantage of the weighted least squares method is that it was developed to handle unbalanced datasets to allow the analysis of all sample data. A disadvantage to the weighted least squares model is if most of the samples of a particular breed did not have the large effect variant at a particular SNP, then that variant may go undetected during analysis because average allelic frequencies are calculated per SNP per breed. There is growing scientific interest in ordinal categorial GWAS approaches optimized for repository SNP data (Bi et al., 2021), and alternative future approaches may yield improved performance. In a recent study that performed LMM analysis with breed phenotypes, breed groups were fixed at *n* = 4 (Plassais et al., 2019). More work is needed to study the effect of variation of breed class number on the association model results.

Defining genome-wide significance dynamically for each model would also be helpful. Bonferroni correction may be too conservative for the weighted least squares method, as supported by the chromosome 16 locus identified from OFA HD SNPs that did not meet genome-wide significance but overlapped with a previous HD candidate locus. Conversely, a more stringent cutoff may be needed for haplotype GWAS to reduce the risk of false positives. FDR correction was not sufficient by itself to dampen the extreme genomic inflation of the haplotype-based analysis. The FDR correction of the LMM did limit the number of significant loci to fewer genomic regions and even had no reported significant loci for the OFA HD dataset.

There were also limitations to the binary case-control GWAS regarding stringency parameters for breed classification as excessively stringent classification reduced sample size. Reduced sample size may increase the likelihood of a false-positive association that reflects a contrast of other breed phenotypes different from the complex disease of interest. This problem should self-correct as the number of genomes in the Dog10K project expands. Overall, the dichotomous case-control GWAS had the smallest number of significant SNPs detected for each disease revealing that information is lost when an ordinal categorical phenotype is arbitrarily made into a dichotomous binary case-control phenotype. Case-control GWAS may allow important large-effect disease-associated genetic variants of breeds considered moderate risk, not classified as high or low risk, to go undetected.

Although SNP and dog filtering was robustly considered, including confirming the phylogeny of individual samples using a cladogram, while aiming to preserve as many dogs as possible in each breed group, further data filtering could have been considered, such as excluding individuals with very low sequencing depth. However, despite differences between HD breed prevalence (Witsberger et al., 2008; Oberbauer et al., 2017) and differences in the number of dogs in each breed in our dataset, we were able to identify both novel and previously reported large effect variants. The hospital HD and ACL rupture datasets consistently had more genomic inflation than the OFA datasets. Excess genomic inflation in the hospital dataset may be attributable to specific breeds containing large numbers of dogs, which was not the case in the OFA dataset.

In conclusion, we investigated four approaches for across-breed GWAS with breed phenotypes for three major canine orthopedic diseases. The use of multiple GWAS methods helped us to detect significant associations. For HD, strong associations with genes that regulate morphology were found, suggesting that the intense selection that gave rise to diverse breeds of dog has also influenced breed risk for HD. We also discovered a strong association between ED and a biologically relevant gene, *FAF1,* that warrants validation. Further investigation is needed to explore alternative methods for haplotype GWAS. Here, use of a moving window to dynamically define haplotype may be beneficial. Further optimization of the methods for the estimation of genome-wide significance appropriate for each model is also needed to appropriately control the false discovery rate. Categorical GWAS of ancestral populations may contribute to the understanding of any disease for which epidemiological risk data are available for the breed or ancestral population, including diseases for which GWAS has not been performed and candidate loci remain elusive.

## Data Availability

The genome sequence accessions and metadata are available from the Dog Biomedical Variant Database Consortium (Jagannathan et al., 2019). The variants are available at the European Variant Archive under project ID PRJEB32865 at https://www.ebi.ac.uk/eva/?eva-study=PRJEB32865. Our SNP set was a newer version of the variants in this repository. The current DBVDC dataset is available by request from the consortium.
