# Supplementary material for: Across-breed genetic investigation of canine hip dysplasia, elbow dysplasia, and anterior cruciate ligament rupture using whole-genome sequencing
Source: Front Genet. 2022 Dec 2;13:913354. doi: 10.3389/fgene.2022.913354 (PMC9755188; doi:10.3389/fgene.2022.913354)
Supplement: Supplementary file 2 [file DataSheet3.docx]

**Table S1**. Summary statistics on 45,165,129 variants and 648 canids in the unfiltered Dog10K VCF.

|  | **Minimum** | **Mean** | **Maximum** |
| --- | --- | --- | --- |
| **Variant Mean Depth** | 0 | 18.8 | 1,541.1 |
| **Variant Missingness (%)** | 0.15 | 7.54 | 99.85 |
| **Variant Quality** | 30 | 43,170 | 25,177,600 |
| **Individual Mean Depth** | 0.005 | 18.88 | 43.19 |
| **Individual Missingness (%)** | 2.23 | 7.54 | 99.88 |

**Table S2.** Phenotypes for binary case-control association for hip dysplasia, elbow dysplasia, and anterior cruciate ligament rupture

|  | Lowest Prevalence | Highest Prevalence | Stringent Scenario | | | | Lenient Scenario | | | |
| --- | --- | --- | --- | --- | --- | --- | --- | --- | --- | --- |
|  |  |  | Controls | | Cases | | Controls | | Cases | |
|  |  |  | Prevalence Cutoff | Number of Dogs | Prevalence Cutoff | Number of Dogs | Prevalence Cutoff | Number of Dogs | Prevalence Cutoff | Number of Dogs |
| OFA HD  (Oberbauer et al., 2017) | 3.74 | 24.75 | ≤8.71 | 43 | ≥16.56 | 39 | ≤10.1 | 79 | ≥13.44 | 59 |
| ED  (Oberbauer et al., 2017) | 0.33 | 48.63 | ≤1.13 | 49 | ≥12.3 | 37 | ≤2.79 | 74 | ≥8.63 | 73 |
| Hospital HD  (Witsberger et al., 2008) | 0.12 | 17.16 | ≤0.23 | 36 | ≥7.37 | 47 | ≤0.62 | 109 | ≥4.23 | 88 |
| ACL rupture  (Witsberger et al., 2008) | 0.21 | 8.9 | ≤0.63 | 39 | ≥2.77 | 40 | ≤1.11 | 66 | ≥2.41 | 65 |

**Note:** OFA, Orthopedic Foundation for Animals; HD, hip dysplasia; ED, elbow dysplasia; ACL, anterior cruciate ligament. Two different cutoff thresholds were tested to determined breeds that would be phenotyped as case or controls. The first stringent scenario prioritized making the cutoff thresholds for disease prevalence’s to be either a case or control more disparate. However, case and controls groups had approximately 40 dogs each due to low representation of breeds of high and low risk for HD, ED, and ACL rupture currently in Dog10K. The second lenient scenario permitted less stringent cutoff thresholds to define a breed as a case or control to prioritize having larger number of individuals per group. The lenient scenario had ~70 dogs per group.

**Table S3.** Breed representation of binary case-control association for OFA hip dysplasia

| **Breed** | **Prevalence of OFA HD (Oberbauer et al., 2017)** | **Number of dogs,**  **OFA HD binary case-control** | |
| --- | --- | --- | --- |
|  |  | **Lenient**  **Scenario** | **Stringent**  **Scenario** |
| Newfoundland | 24.75 | 2 | 2 |
| American Staffordshire Terrier | 24.39 | 2 | 2 |
| Bullmastiff | 23.97 | 2 | 2 |
| Rottweiler | 20.07 | 3 | 3 |
| Chow Chow | 19.17 | 1 | 1 |
| German Shepherd | 18.94 | 16 | 16 |
| Golden Retriever | 18.84 | 10 | 10 |
| Old English Sheepdog | 17.76 | 1 | 1 |
| Pembroke Welsh Corgi | 16.56 | 2 | 2 |
| Greater Swiss Mountain Dog | 15.38 | 6 | - |
| Tibetan Mastiff | 14.26 | 9 | - |
| Australian Cattle Dog | 13.44 | 5 | - |
| Leonberger | 12.72 | - | - |
| Great Dane | 11.62 | - | - |
| Cavalier King Charles Spaniel | 11.35 | - | - |
| Labrador Retriever | 11.24 | - | - |
| Standard Poodle | 10.99 | - | - |
| Portuguese Water Dog | 10.98 | - | - |
| Alaskan Malamute | 10.83 | - | - |
| Welsh Springer Spaniel | 10.8 | - | - |
| Boxer | 10.7 | - | - |
| Border Collie | 10.1 | 36 | - |
| Great Pyrenees | 8.71 | 1 | 1 |
| German Wirehaired Pointer | 8.02 | 1 | 1 |
| Weimaraner | 7.94 | 2 | 2 |
| Havanese | 6.25 | 3 | 3 |
| Vizsla | 6.19 | 2 | 2 |
| Bichon Frise | 5.88 | 4 | 4 |
| Nova Scotia Duck Tolling Retriever | 5.81 | 1 | 1 |
| Doberman Pinscher | 5.67 | 3 | 3 |
| Australian Shepherd | 5.33 | 2 | 2 |
| Bearded Collie | 5.3 | 8 | 8 |
| Belgian Malinois | 5.16 | 5 | 5 |
| Irish Wolfhound | 4.47 | 2 | 2 |
| Rhodesian Ridgeback | 4.42 | 4 | 4 |
| Shetland Sheepdog | 4.16 | 3 | 3 |
| Flat Coated Retriever | 3.74 | 2 | 2 |
| **Case** |  | **59** | **39** |
| **Control** |  | **79** | **43** |
| **Total** |  | **138** | **82** |

**Note:** OFA, Orthopedic Foundation for Animals; HD, hip dysplasia.

**Table S4.** Breed representation of binary case-control association for elbow dysplasia

| **Breed** | **Prevalence of OFA ED (Oberbauer et al., 2017)** | **Number of dogs,**  **ED binary case-control** | |
| --- | --- | --- | --- |
|  |  | **Lenient**  **Scenario** | **Stringent**  **Scenario** |
| Chow Chow | 48.63 | 1 | 1 |
| Rottweiler | 38.07 | 3 | 3 |
| Newfoundland | 22.7 | 2 | 2 |
| German Shepherd | 17.83 | 16 | 16 |
| American Staffordshire Terrier | 16.07 | 2 | 2 |
| Bullmastiff | 14.2 | 2 | 2 |
| Tibetan Mastiff | 13.84 | 9 | 9 |
| Irish Wolfhound | 12.3 | 2 | 2 |
| Australian Cattle Dog | 9.8 | 5 | - |
| Golden Retriever | 9.69 | 10 | - |
| Labrador Retriever | 9.57 | 10 | - |
| Greater Swiss Mountain Dog | 9.11 | 6 | - |
| Belgian Malinois | 8.63 | 5 | - |
| Havanese | 5.46 | - | - |
| Rhodesian Ridgeback | 4.95 | - | - |
| Great Dane | 3.53 | - | - |
| Australian Shepherd | 3.5 | - | - |
| Old English Sheepdog | 3.36 | - | - |
| Leonberger | 3.25 | - | - |
| Alaskan Malamute | 2.97 | - | - |
| Pembroke Welsh Corgi | 2.92 | - | - |
| Standard Poodle | 2.81 | - | - |
| Shetland Sheepdog | 2.79 | 3 | - |
| Nova Scotia Duck Tolling Retriever | 2.51 | 1 | - |
| German Wirehaired Pointer | 2.17 | 1 | - |
| Vizsla | 1.99 | 2 | - |
| Bearded Collie | 1.94 | 8 | - |
| Weimaraner | 1.57 | 2 | - |
| Great Pyrenees | 1.45 | 1 | - |
| Welsh Springer Spaniel | 1.44 | 4 | - |
| Portuguese Water Dog | 1.43 | 3 | - |
| Border Collie | 1.13 | 36 | 36 |
| Doberman Pinscher | 0.82 | 3 | 3 |
| Boxer | 0.73 | 1 | 1 |
| Flat Coated Retriever | 0.68 | 2 | 2 |
| Bichon Frise | 0.45 | 4 | 4 |
| Cavalier King Charles Spaniel | 0.33 | 3 | 3 |
| **Case** |  | **73** | **37** |
| **Control** |  | **74** | **49** |
| **Total** |  | **147** | **86** |

**Note:** OFA, Orthopedic Foundation for Animals; ED, elbow dysplasia;

**Table S5.** Breed representation of binary case-control association for hospital hip dysplasia

| **Breed** | **Prevalence of hospital HD (Witsberger et al., 2008)** | **Number of dogs,**  **hospital HD binary case-control** | |
| --- | --- | --- | --- |
|  |  | **Lenient**  **Scenario** | **Stringent**  **Scenario** |
| Newfoundland | 17.16 | 2 | 2 |
| Saint Bernard | 14.7 | 2 | 2 |
| Old English Sheepdog | 11.1 | 1 | 1 |
| Rottweiler | 10.53 | 3 | 3 |
| German Shepherd | 10.26 | 16 | 16 |
| Golden Retriever | 8.51 | 10 | 10 |
| Alaskan Malamute | 7.8 | 3 | 3 |
| Labrador Retriever | 7.37 | 10 | 10 |
| Chow Chow | 6.44 | 1 | - |
| Airedale Terrier | 6.22 | 3 | - |
| Bulldog | 4.42 | 1 | - |
| Border Collie | 4.23 | 36 | - |
| Great Dane | 3.89 | - | - |
| Weimaraner | 3.39 | - | - |
| Australian Shepherd | 3.06 | - | - |
| Standard Poodle | 2.79 | - | - |
| Boxer | 2.12 | - | - |
| Siberian Husky | 2.01 | - | - |
| American Staffordshire Terrier | 1.84 | - | - |
| Shetland Sheepdog | 1.83 | - | - |
| Basset Hound | 1.6 | - | - |
| Pug | 1.53 | - | - |
| Dalmatian | 1.36 | - | - |
| Collie | 1.34 | - | - |
| Doberman Pinscher | 1.34 | - | - |
| American Cocker Spaniel | 0.87 | - | - |
| Shih Tzu | 0.73 | - | - |
| West Highland White Terrier | 0.64 | - | - |
| Beagle | 0.62 | 5 | - |
| Pomeranian | 0.59 | 3 | - |
| Miniature Poodle | 0.51 | 1 | - |
| Greyhound | 0.37 | 5 | - |
| Yorkshire Terrier | 0.36 | 59 | - |
| Chihuahua | 0.23 | 4 | 4 |
| Miniature Schnauzer | 0.2 | 24 | 24 |
| Miniature Dachshund | 0.17 | 3 | 3 |
| Dachshund | 0.15 | 4 | 4 |
| Scottish Terrier | 0.12 | 1 | 1 |
| **Case** |  | **88** | **47** |
| **Control** |  | **109** | **36** |
| **Total** |  | **197** | **83** |

**Note:** HD, hip dysplasia.

**Table S6.** Breed representation of binary case-control association for anterior cruciate ligament rupture

| **Breed** | **Prevalence of hospital ACL rupture (Witsberger et al., 2008)** | **Number of dogs,**  **ACL rupture binary case-control** | |
| --- | --- | --- | --- |
|  |  | **Lenient**  **Scenario** | **Stringent**  **Scenario** |
| Newfoundland | 8.9 | 2 | 2 |
| Rottweiler | 8.29 | 3 | 3 |
| Labrador Retriever | 5.79 | 10 | 10 |
| Bulldog | 5.33 | 1 | 1 |
| Boxer | 5.24 | 1 | 1 |
| Chow Chow | 4.3 | 1 | 1 |
| American Staffordshire Terrier | 4.05 | 2 | 2 |
| Saint Bernard | 3.57 | 2 | 2 |
| Alaskan Malamute | 3.25 | 3 | 3 |
| Airedale Terrier | 3.22 | 3 | 3 |
| Miniature Poodle | 2.95 | 1 | 1 |
| Golden Retriever | 2.8 | 10 | 10 |
| Great Dane | 2.77 | 1 | 1 |
| West Highland White Terrier | 2.73 | 19 | - |
| Dalmatian | 2.72 | 1 | - |
| Beagle | 2.41 | 5 | - |
| American Cocker Spaniel | 2.36 | - | - |
| Yorkshire Terrier | 2.27 | - | - |
| German Shepherd | 2.24 | - | - |
| Australian Shepherd | 2.14 | - | - |
| Siberian Husky | 2.12 | - | - |
| Chihuahua | 2.04 | - | - |
| Border Collie | 2 | - | - |
| Standard Poodle | 1.87 | - | - |
| Doberman Pinscher | 1.85 | - | - |
| Weimaraner | 1.77 | - | - |
| Pomeranian | 1.43 | - | - |
| Shetland Sheepdog | 1.34 | - | - |
| Scottish Terrier | 1.32 | - | - |
| Basset Hound | 1.11 | 5 | - |
| Old English Sheepdog | 0.97 | 1 | - |
| Collie | 0.76 | 1 | - |
| Pug | 0.75 | 20 | - |
| Miniature Schnauzer | 0.63 | 24 | 24 |
| Shih Tzu | 0.57 | 3 | 3 |
| Greyhound | 0.55 | 5 | 5 |
| Dachshund | 0.21 | 4 | 4 |
| Miniature Dachshund | 0.21 | 3 | 3 |
| **Case** |  | **65** | **40** |
| **Control** |  | **66** | **39** |
| **Total** |  | **131** | **79** |

**Note:** ACL, anterior cruciate ligament.
