# Supplementary material for: Across-breed genetic investigation of canine hip dysplasia, elbow dysplasia, and anterior cruciate ligament rupture using whole-genome sequencing
Source: Front Genet. 2022 Dec 2;13:913354. doi: 10.3389/fgene.2022.913354 (PMC9755188; doi:10.3389/fgene.2022.913354)
Supplement: Supplementary file 3 [file DataSheet2.docx]

**Supplementary Figures**


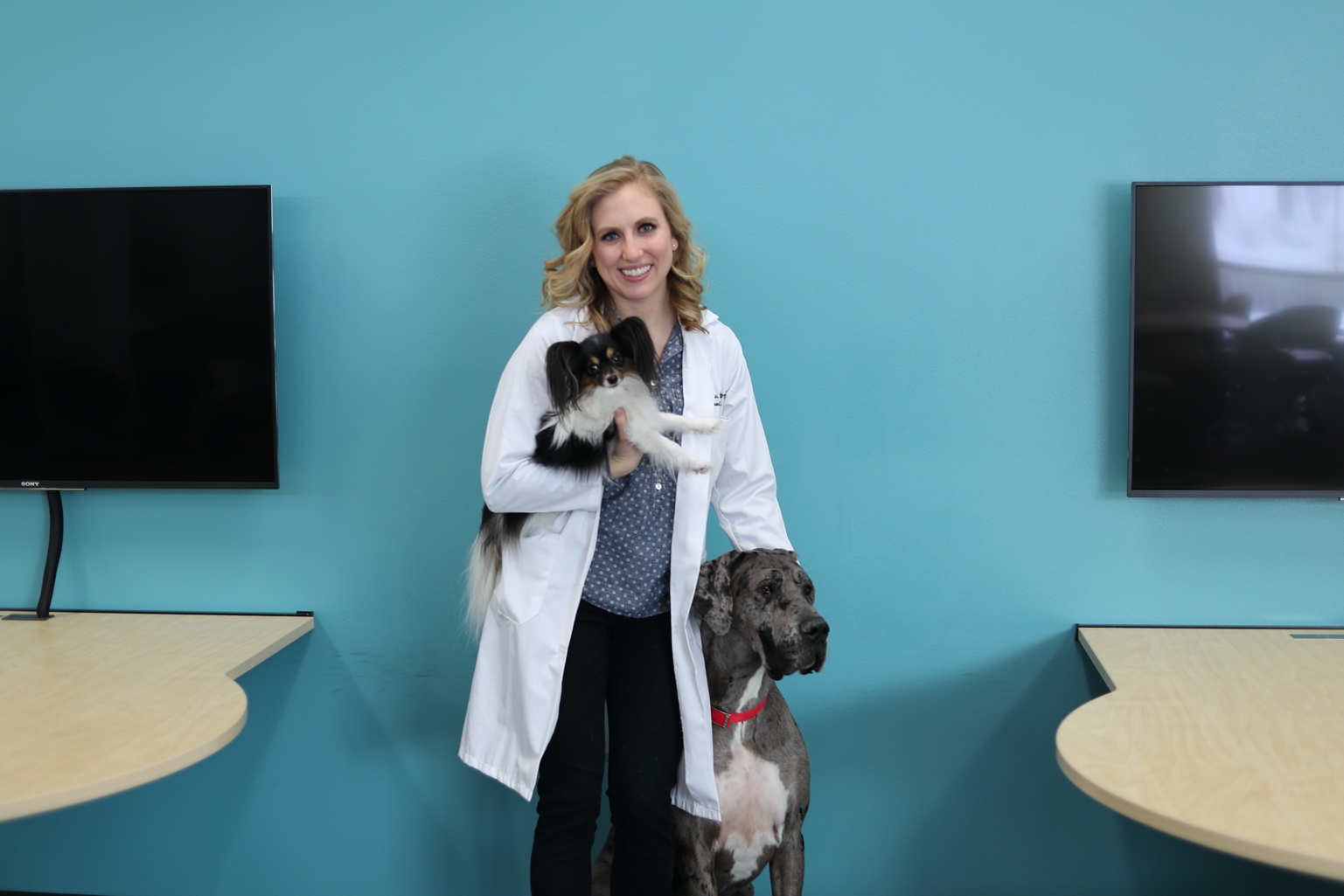
**Figure S1.** **The modern dog is one of the most phenotypically diverse domesticated species**. In dogs, distinctive breed defining morphological phenotypes, such as withers height, have been under strong artificial selection which has likely increased the prevalence of large effect variants underlying these breed defining traits (Hayward et al., 2016). Domestic dog breeds display some of the most extreme phenotypic diversity as demonstrated by overall size differences between a Great Dane and Papillon. Differences between body size of the largest and smallest breeds can differ almost 40-fold. Strong artificial selection and population bottlenecks during canine domestication has created divergent breed defining behavioral and morphologic traits but has also, inadvertently, resulted in enrichment of disease associated variants within certain breeds. For many complex polygenic canine diseases considerable differences in disease risk exist across breeds. Disease associated risk variants that have increased in frequency or become fixed within a breed enable successful across-breed genome-wide association studies (GWASs) to be performed using breed average (summary level) phenotype information for discovery of large effect disease associated variants.


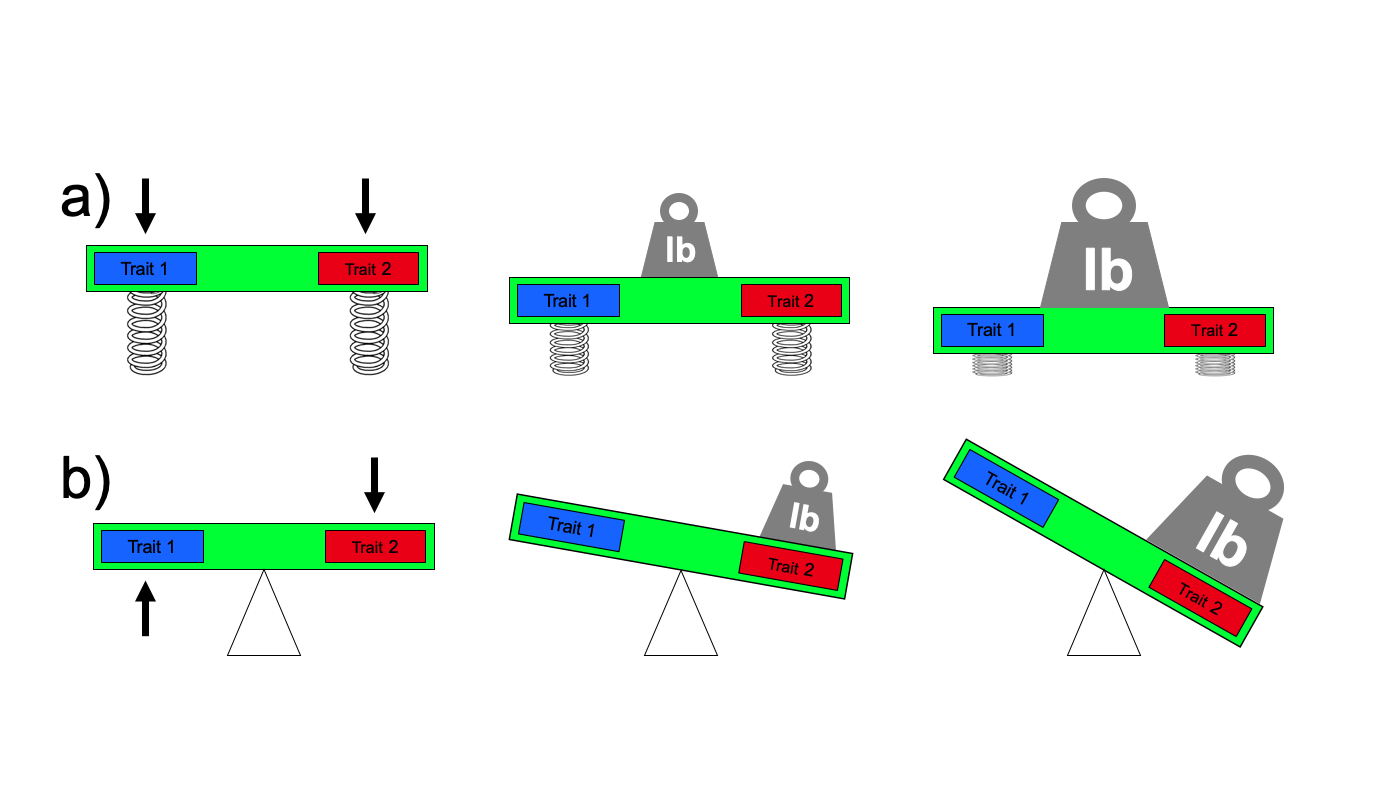
**Figure S2.** **Genetic correlation between two traits**. Genetic correlation is the quantitative measurement of both direction and magnitude of the association of genotypes of two traits (Brenner et al., 2002). Directionality of the association can either be (**a**) in the same direction or (**b**) in opposite directions. If genetic correlation is zero, then the genotype of one trait is not associated with the genotype of another trait implying both traits are independent of each other (Cheverud 1988). Genetic correlation can be caused by linkage disequilibrium and pleiotropy (Cheverud 1988; Breed and Moore 2010; Bolormaa et al., 2014). Linkage disequilibrium is the non-random association of alleles at different loci. Pleiotropy is the phenomenon that one causal genetic variant can affect multiple phenotypic traits. During canine domestication, selective breeding for certain morphological or behavioral traits (i.e., chondrodysplasia, brachycephaly, herding, retrieving) may have inadvertently increased frequency of disease associated risk variants through genetic correlation (Sargan 2004; Karlsson and Lindblad-Toh 2008).

**Figure S3.** **Differences between complex trait investigation using across-breed GWAS using summary level phenotypes compared to within-breed GWAS using individual level phenotypes**. Modern dog breeds display some of the most extreme phenotypic diversity of mammals. Years of strong artificial selection and population bottlenecks have resulted in distinct closed breeding populations (breeds) with fixated extreme phenotypes (Parker et al., 2009). Intense artificial selection has inadvertently increased frequency and fixation of undesirable disease associated risk variants in certain breeds (Parker et al., 2009). As a result, certain complex polygenic canine diseases have a wide variation of prevalence (occurrence) between breeds. Genetic dissection of canine complex polygenic traits and diseases can be performed by both across-breed and (**b**) within-breed genome-wide association (GWAS) using individual level phenotype information. However, when individual phenotypes are lacking genetic dissection can still be performed with (**a**) across-breed GWAS using summary level (breed average) phenotype information because of the unique evolutionary history of the modern dog. (**a**) Across-breed GWAS with breed average phenotype information can detect fixed large effect genetic variants that are primarily responsible for large differences across breeds but fails to detect small to moderate effect variants that are responsible for within-breed phenotypic heterogeneity (Hayward et al., 2016; Bannasch et al., 2020). (**b**) Within-breed GWAS with individual phenotyping is able to detect numerous small to moderate effect loci that are responsible for within breed phenotypic diversity (Hayward et al., 2016; Bannasch et al., 2020).

**
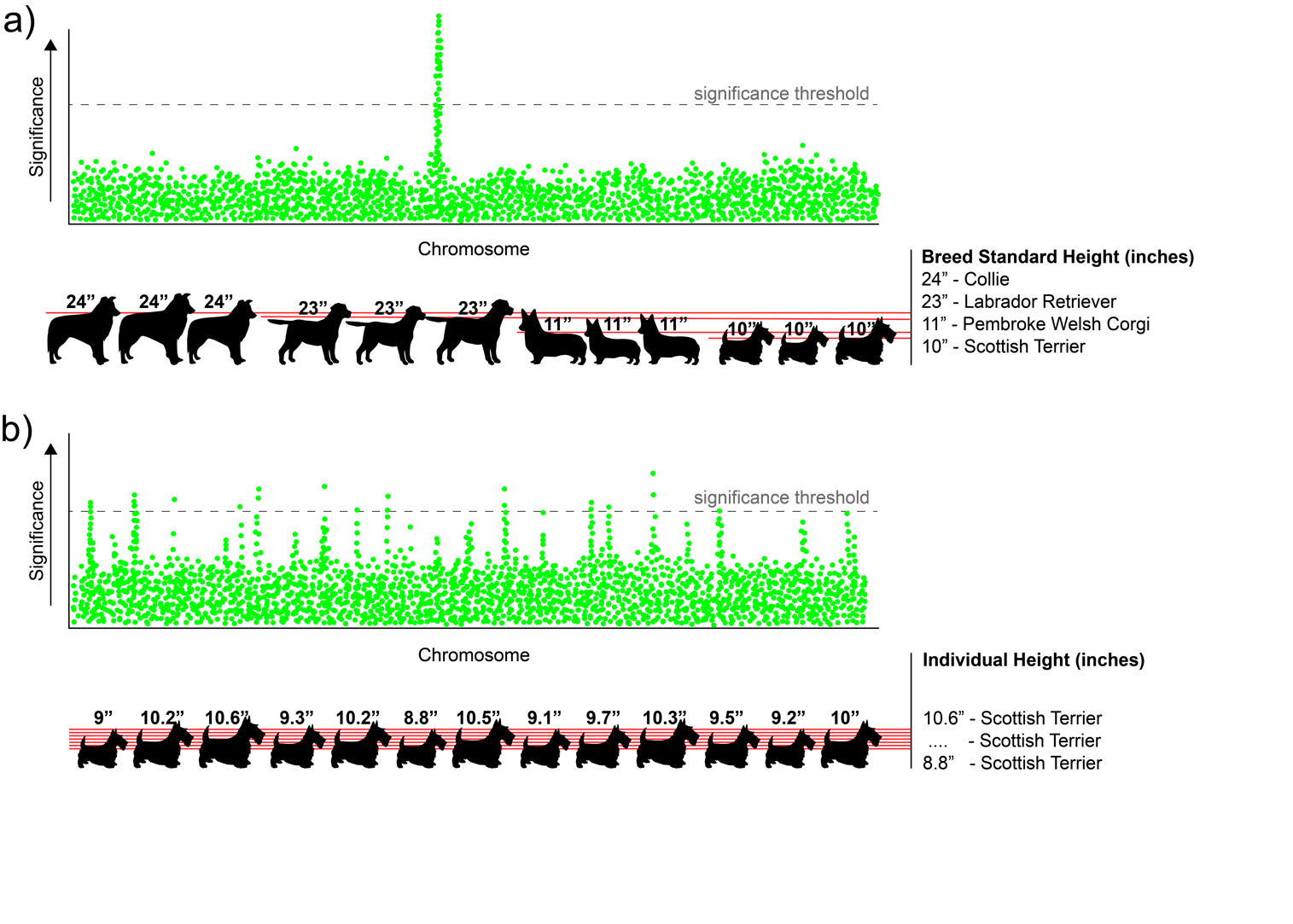
**

**Figure S4. Graphical summary statistics on variants and samples on VCF file with 648 individuals.** (**1a**) Plot of variant (site) and (**1b**) individual (sample) mean depth. (**2a**) Plot of variant (site) and (**2b**) individual (sample) missingness. All quality analysis on the raw VCF obtained from Dog10K was performed using vcftools (Danecek et al., 2011) and tidyverse in R (Wickham et al., 2019).


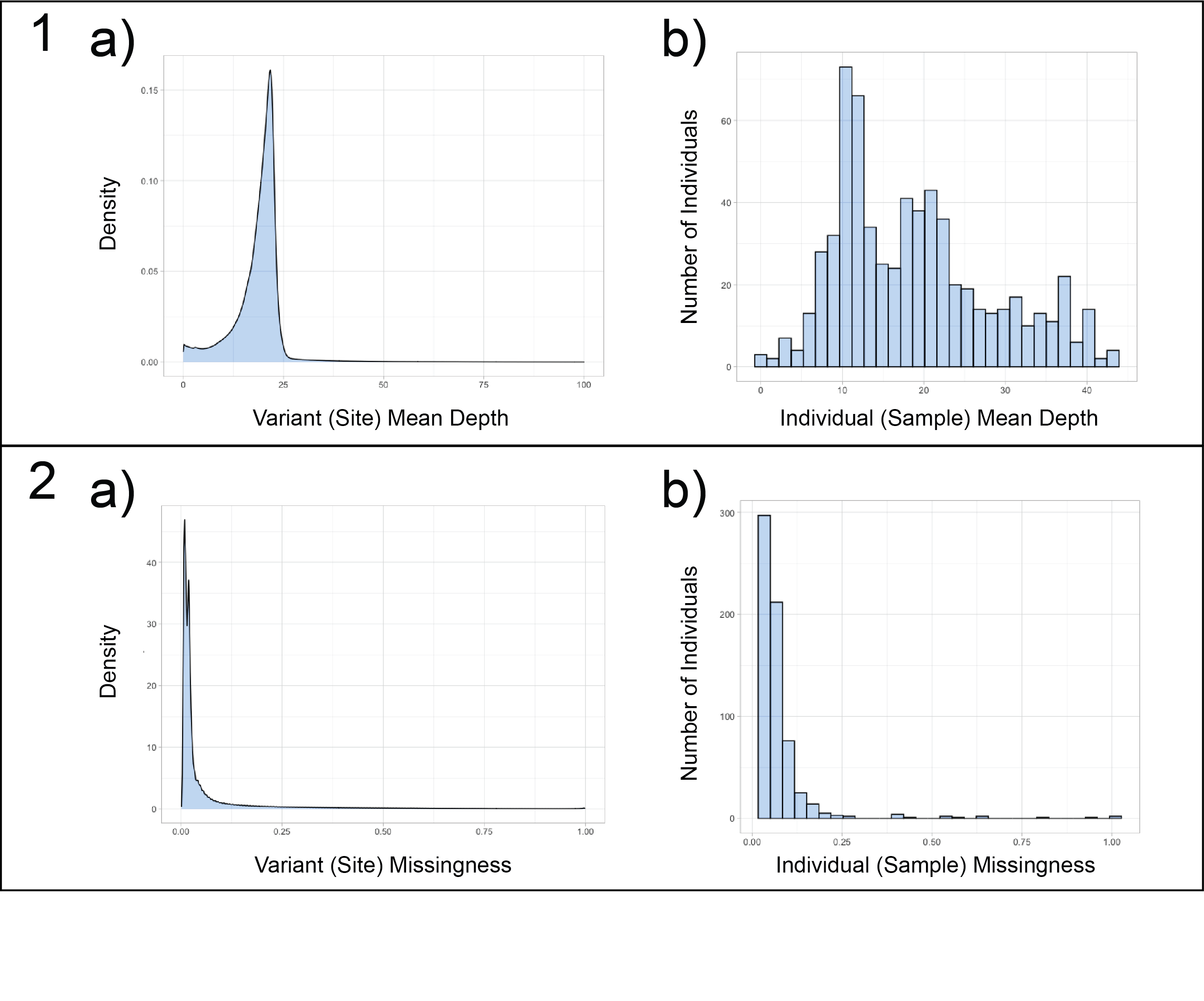


**Figure S5.** **Cladogram of 1,672 dogs.** To detect Dog10K samples with discordant breed information we performed neighbor joining phylogeny. We compared the 562 domestic dogs with WGS data to 1,110 canids with SNP array data that was previously published (accession: GSE123368, GSE70454, GSE83160, GSE90441, and GSE96736) (Vaysse et al., 2011; Decker et al., 2015; Dreger et al., 2016ab; Parker et al., 2017; Plassais et al., 2019). A total of 12 individuals were found to be discordant and were removed from analysis.


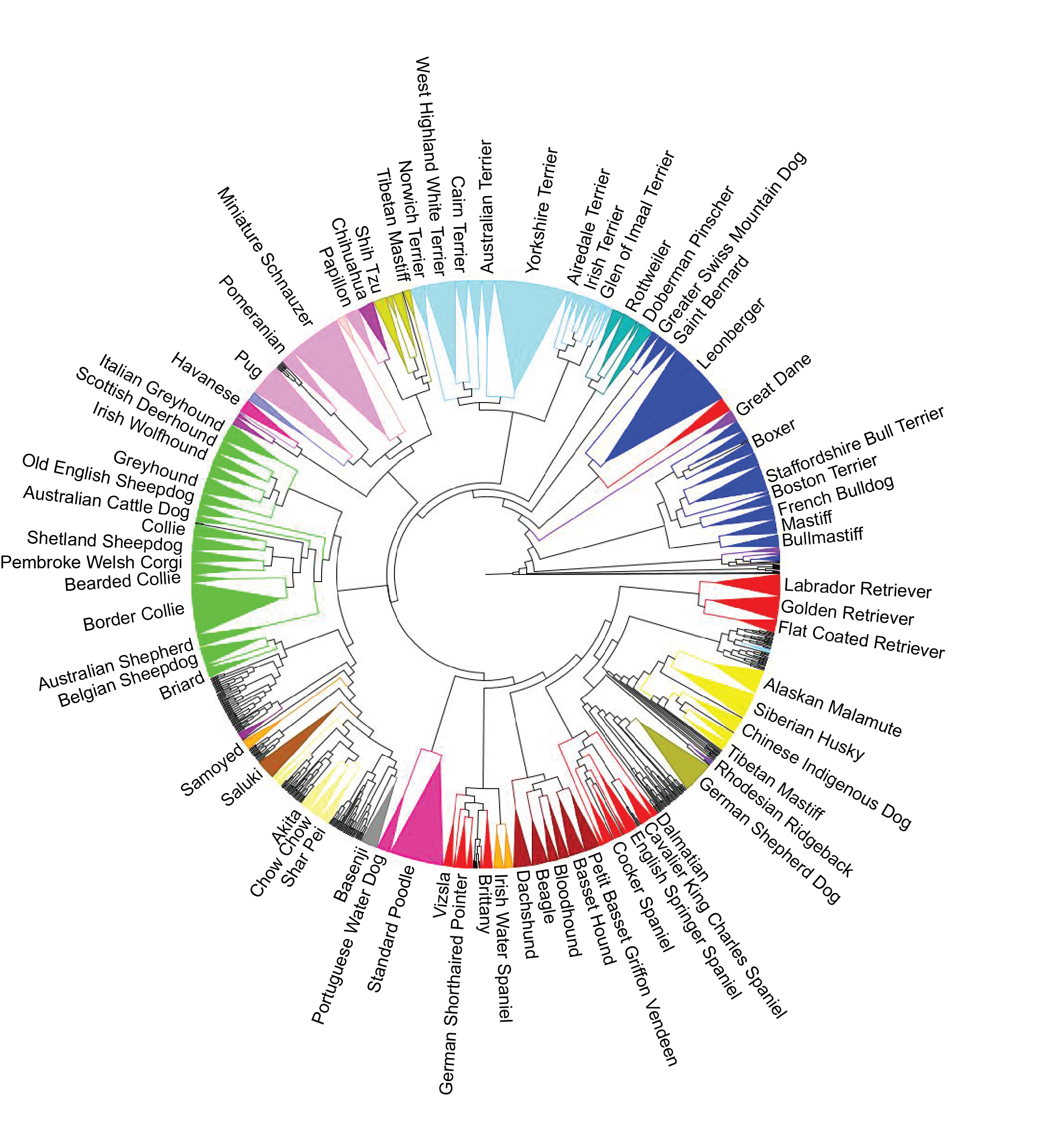


**Figure S6.** **Manhattan plots of false discovery rate correction of linear mixed model across-breed GWAS using breed average phenotypes for HD, ED, and ACL rupture**. *P* value adjustment was performed using false discovery rate (FDR) correction with fdrtool in R (Strimmer 2008) for linear mixed model association analysis for hip dysplasia (HD), elbow dysplasia (ED), and anterior cruciate ligament (ACL) rupture. Plots (**a**) and (**b**) represent analysis of 7,586,942 single nucleotide polymorphisms (SNPs) from 230 dogs from 27 breeds with assigned phenotypes of breed disease prevalence for HD and ED respectively as reported in a publication using OFA data (Oberbauer et al., 2017). Plots (**c**) and (**d**) represent analysis of 7,026,774 SNPs from 279 dogs from 38 breed with assigned phenotypes of breed disease prevalence for HD and ACL rupture, respectively, as reported by a publication using veterinary hospital data (Witsberger et al., 2008). (**a**) After FDR correction no significant loci were found at *P*<6.59E-9 for the OFA HD analysis. (**b**) After FDR correction 83 loci were found at *P*<6.59E-9 for the OFA ED analysis with the most significant locus located on chromosome 24 and other significant loci on chromosomes 1, 2, 8, 15, 16, and 33. (**c**) The hospital HD dataset had 11 significant loci at *P*<7.129E-9 located on chromosome 3 and 17 after FDR correction. (**d**) After FDR correction, the hospital ACL rupture analysis had 47 significant loci at *P*<7.129E-9 located on chromosome 17 and 24.


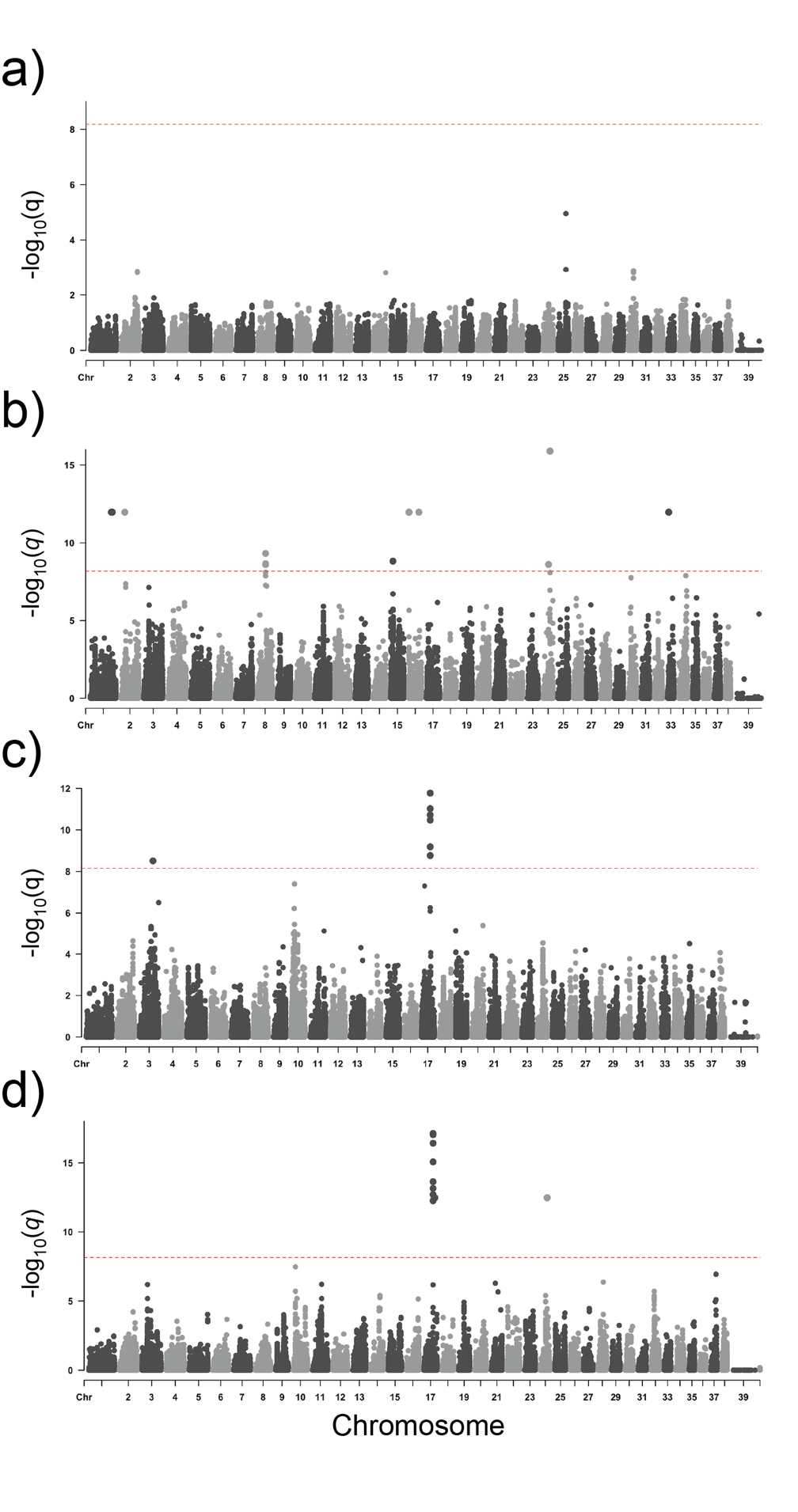


**
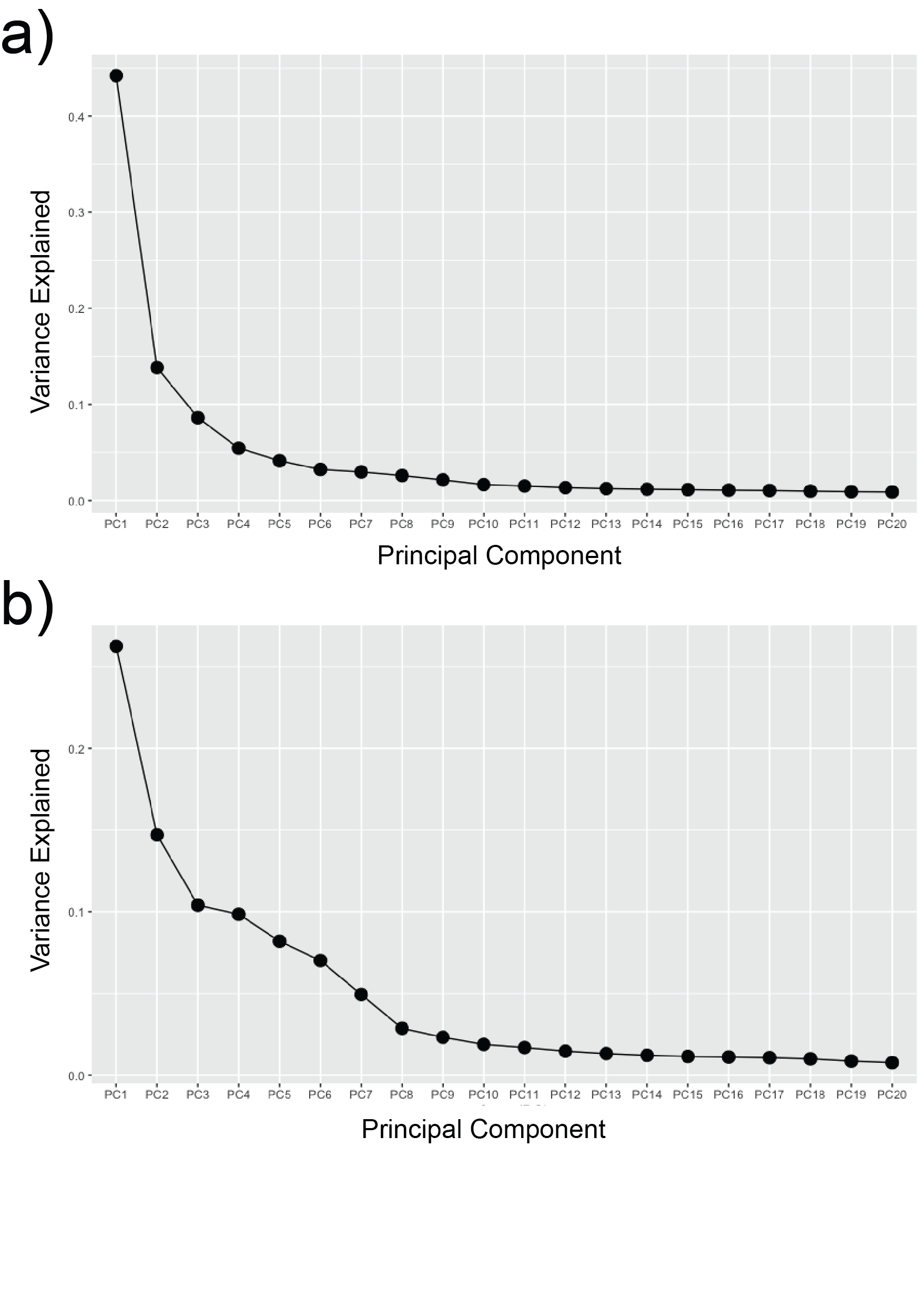
Figure S7.** **Scree plot of principal components eigenvectors for OFA HD, ED, hospital HD and ACL rupture**. Scree plots were draw from the generated centered relatedness matrix made with GEMMA v0.94.1 (Zhou and Stephens 2012). (**a**) Scree plot for the Orthopedic Foundation for Animals (OFA) hip dysplasia (HD) and elbow dysplasia (ED) datasets. (**b**) Scree plot for the hospital HD and anterior cruciate ligament (ACL) rupture datasets. For haplotype-based genome wide association study (GWAS) the first 6 eigenvectors and the first 8 eigenvectors were incorporated as covariates for the OFA HD and ED analysis and the hospital HD and ACL rupture datasets, respectively.

**Figure S8.** **Manhattan plots of false discovery rate correction of haplotype-based model across-breed GWAS using breed average phenotypes for HD, ED, and ACL rupture**. *P* value adjustment was performed using false discovery rate (FDR) correction with fdrtool in R (Strimmer 2008) for haplotype-based model association analysis for hip dysplasia (HD), elbow dysplasia (ED), and anterior cruciate ligament rupture (ACL) rupture. Plots (**a**) and (**b**) represent analysis of 7,586,822 sliding windows with a fixed width of 4 single nucleotide polymorphisms (SNPs) from 230 dogs from 27 breeds with assigned phenotypes of breed disease prevalence for HD and ED respectively as reported in a publication using OFA data (Oberbauer et al., 2017). Plots (**c**) and (**d**) represent analysis of 7,026,651 sliding windows of fixed 4 SNP width from 279 dogs from 38 breed with assigned phenotypes of breed disease prevalence for HD and ACL rupture, respectively, as reported by a publication using veterinary hospital data (Witsberger et al., 2008). (**a**) After FDR correction 36 significant loci were found at *P*<1.68E-9 for the OFA HD analysis with the most significant loci on chromosome 25 and other significant loci on 1, 2, 8, 21 and 34. (**b**) After FDR correction 23,598 loci were found at *P*<1.68E-9 for the OFA ED analysis with the most significant locus located on chromosome 15 and some of the other significant loci on chromosomes 19, 30, 31, 33 and 35. (**c**) The hospital HD dataset had 4,294 significant loci at *P*<1.82E-9 with the most significant loci located on chromosome 17 and some others on 3, 5, 7, 22, 26, 27 after FDR correction. (**d**) After FDR correction, the hospital ACL rupture analysis had 10,403 significant loci at *P*<1.82E-9 located on chromosome 17 and some others on 19, 21, 23, 25, 32, 33.


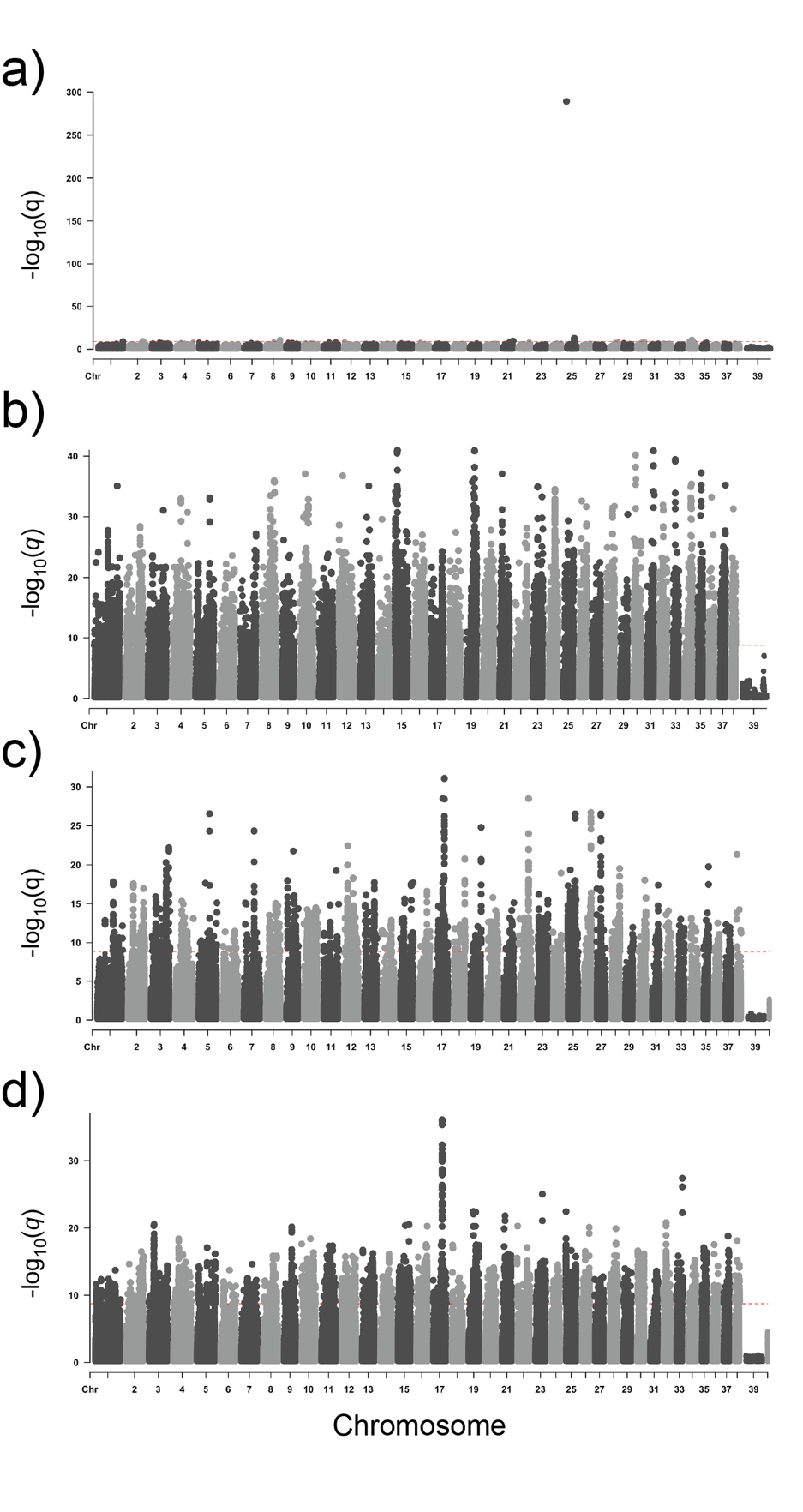


**Supplementary References**

Bannasch, D.L., Baes, C.F., Leeb, T. (2020). Genetic variants affecting skeletal morphology in domestic dogs. *Trends Genet*. 36, 598-609. doi: 10.1016/j.tig.2020.05.005

Bolormaa, S., Pryce, J.E., Reverter, A., Zhang, Y., Barendse, W., Kemper, K., et al. (2014). A multi-trait, meta-analysis for detecting pleiotropic polymorphisms for stature, fatness and reproduction in beef cattle. *PLoS Genetics*. 10, e1004198. doi: 10.1371/journal.pgen.1004198

Breed, M.D., Moore, J. (2010). Encyclopedia of Animal Behavior. Cambridge, MA: Academic Press.

Brenner, S., Miller, J.H., Broughton, W. (2002). Encyclopedia of Genetics. Cambridge, MA: Academic Press.

Cheverud, J.M. (1988). A comparison of genetic and phenotypic correlations. *Evolution*. 42, 958-968. doi: 10.1111/j.1558-5646

Danecek, P., Auton, A., Abecasis, G., Albers, C.A., Banks, E., DePristo, M.A., et al. (2011). The variant call format and VCFtools. *Bioinformatics*. 27, 2156-2158. doi: 10.1093/bioinformatics/btr330

Decker, B., Davis, B.W., Rimbault, M., Long, A.H., Karlins, E., Jagannathan, V., et al. (2015). Comparison against 186 canid whole-genome sequences reveals survival strategies of an ancient clonally transmissible canine tumor. *Genome Res*. 25, 1646-1655. doi: 10.1101/gr.190314.115

Dreger, D.L., Davis, B.W., Cocco, R., Sechi, S., Di Cerbo, A., Parker, H.G., et al. (2016a). Commonalities in development of pure breeds and population isolates revealed in the genome of the Sardinian Fonni's Dog. *Genetics*. 204, 737-755. doi: 10.1534/genetics.116.192427

Dreger, D.L., Rimbault, M., Davis, B.W., Bhatnagar, A., Parker, H.G., Ostrander, E.A. (2016b). Whole-genome sequence, SNP chips and pedigree structure: building demographic profiles in domestic dog breeds to optimize genetic-trait mapping. *Dis Model Mech*. 9, 1445-1460. doi: 10.1242/dmm.027037

Hayward, J.J., Castelhano, M.G., Oliveira, K.C., Corey, E., Balkman, C., Baxter, T.L., et al. (2016). Complex disease and phenotype mapping in the domestic dog. *Nature Commun*. 7, 10460. doi: 10.1038/ncomms10460

Karlsson, E.K., Lindblad-Toh, K. (2008). Leader of the pack: gene mapping in dogs and other model organisms. *Nature Reviews Genetics*. 9, 713-725. doi: 10.1038/nrg2382

Oberbauer, A.M., Keller, G.G., Famula, T.R. (2017). Long-term genetic selection reduced prevalence of hip and elbow dysplasia in 60 dog breeds. *PLoS One*. 12, e0172918. doi: 10.1371/journal.pone.0172918

Parker, H.G., Dreger, D.L., Rimbault, M., Davis, B.W., Mullen, A.B., Carpintero-Ramirez, G., et al. (2017). Genomic analyses reveal the influence of geographic origin, migration, and hybridization on modern dog breed development. *Cell Rep*. 19, 697-708. doi: 10.1016/j.celrep.2017.03.079

Parker, H.G., VonHoldt, B.M., Quignon, P., Margulies, E.H., Shao, S., Mosher, D.S., et al. (2009). An expressed fgf4 retrogene is associated with breed-defining chondrodysplasia in domestic dogs. *Science*. 325, 995-998. doi: 10.1126/science.1173275

Plassais, J., Kim, J., Davis, B.W., Karyadi, D.M., Hogan, A.N., Harris, A.C., et al. (2019). Whole genome sequencing of canids reveals genomic regions under selection and variants influencing morphology. *Nature Commun*. 10, 1-4. doi: 10.1038/s41467-019-09373-w

Sargan, D.R. (2004). IDID: inherited diseases in dogs: web-based information for canine inherited disease genetics. *Mamm Genome*. 15, 503-506. doi: 10.1007/s00335-004-3047-z

Strimmer, K. (2008). fdrtool: a versatile R package for estimating local and tail area-based false discovery rates. *Bioinformatics*. 24, 1461-1462. doi: 10.1093/bioinformatics/btn209

Vaysse, A., Ratnakumar, A., Derrien, T., Axelsson, E., Pielberg, G.R., Sigurdsson, S., et al. (2011). Identification of genomic regions associated with phenotypic variation between dog breeds using selection mapping. *PLoS Genet*. 7, e1002316. doi: 10.1371/journal.pgen.1002316

Wickham, H., Averick, M., Bryan, J., Chang, W., McGowan, L.D., François, R., et al. (2019). Welcome to the Tidyverse. *J Open Source Softw*. 4, 1686. doi: 10.21105/joss.01686

Witsberger, T.H., Villamil, J.A., Schultz, L.G., Hahn, A.W., Cook, J.L. (2008). Prevalence of and risk factors for hip dysplasia and cranial cruciate ligament deficiency in dogs. *J Am Vet Med Assoc*. 232, 1818-1824. doi: 10.2460/javma.232.12.1818

Zhou, X., Stephens, M. (2012). Genome-wide efficient mixed-model analysis for association studies. *Nature Genet*. 44, 821-824. doi: 10.1038/ng.2310
