# Supplementary material for: Across-breed genetic investigation of canine hip dysplasia, elbow dysplasia, and anterior cruciate ligament rupture using whole-genome sequencing
Source: Front Genet. 2022 Dec 2;13:913354. doi: 10.3389/fgene.2022.913354 (PMC9755188; doi:10.3389/fgene.2022.913354)
Supplement: Supplementary file 5 [file DataSheet1.docx]

**Supplementary Background**

**Hip dysplasia**

Dogs that develop hip dysplasia (HD) are born with normal hips which subsequently become dysplastic. Both genetic and environmental factors contribute to hip joint development and influence joint laxity, subluxation, and conformational changes to the acetabulum and femoral head (Krotscheck and Todhunter 2010). Increased hip laxity, defined as pathologic instability, allows abnormal joint wear which can lead to osteoarthritis (King 2017). The exact causes of HD and hip joint laxity remain unclear. Although hip laxity is a risk factor for the development of HD, hip laxity by itself is not sufficient to cause HD (Lust et al., 1993). Environmental factors such as housing, exercise, body weight and nutrition influence development, presentation, and severity of HD (Richardson 1992; Krontveit et al., 2012). Disease progression and clinical presentation of dogs with HD is variable (Ginja et al., 2009). Dogs typically present as immature dogs with hip laxity and pain caused by stretching of the round ligament, synovitis and acetabular microfractures, or they present as adults with pain from osteoarthritis (Ginja et al., 2010). Clinical signs can include a stiff pelvic limb gait, bunny hopping, shortened stride length, and difficulty climbing stairs.

Standardized radiographic screening methods to evaluate hip conformation and laxity phenotypes can approximate an individual dog’s genetic risk (Smith et al., 1998). HD is most prevalent in large, rapidly growing breeds such as the Newfoundland, Saint Bernard, Rottweiler, German Shepherd Dog, Labrador Retriever, Golden Retriever, and Bernese Mountain Dog (Witsberger et al., 2008).

Numerous studies have investigated the genetic contribution to HD disease risk. Although reported quantitative trait locus associations and candidate genes have not been consistent between studies, the current consensus is that HD is polygenic (Janutta et al., 2006; Zhu et al., 2009; Pfahler and Distl 2012; Sánchez-Molano et al., 2014; Fels and Distl 2014). Inconsistency in HD genetic study results is attributed to differences in study populations and research methods. Studies using variance estimates and Bayesian modeling have provided some evidence that across-breed shared large effect variants for canine HD exist (Hamann et al., 2003; Todhunter et al., 2003; Mäki et al., 2004; Janutta et al., 2006).

**Elbow dysplasia**

Elbow dysplasia (ED) is an umbrella term that represents a group of disorders that describe abnormal elbow joint development which include ununited anconeal process, fragmented medial coronoid process, osteochondritis dissecans of the distal humerus, articular cartilage injury and incongruity of the elbow (Michelsen 2013). All these abnormalities affect the elbow’s articular surfaces. Environmental factors such as nutrition and exercise can influence risk of ED (Nap 1995). Some dogs with ED may have minimal clinical signs, making diagnosis of early disease difficult, particularly in young dogs. Older dogs typically present over the age of 6 years old with advanced osteoarthritis (Vermote et al., 2010). Dogs that have ED with obvious clinical signs at a young age can present with thoracic limb lameness as young as 6 months old. Affected limbs may have a short stiff stride while walking, as well as decreased elbow range of motion and pain on flexion, extension, and lateral rotation.

In addition to an orthopaedic exam, patients may need arthroscopy or advanced diagnostic imaging to determine the underlying pathology. Treatments for ED vary depending on which specific elbow pathology is present. There are no curative procedures for ED. Although both surgical and medical treatments aim to improve joint function, development of osteoarthritis is typical. ED is commonly seen in large breed dogs such as the German Shepherd Dog, Labrador Retriever, Golden Retriever, and Bernese Mountain Dog (Huang et al., 2017). ED is also common in chondrodystrophic breeds like the French Bulldog (Michelsen 2013).

**Anterior cruciate ligament rupture**

Anterior cruciate ligament (ACL) rupture is a degenerative and inflammatory condition with fiber tearing developing in the presence of knee synovitis (Bleedorn et al., 2011). Tearing typically progresses to complete rupture during habitual activity. Dogs with incomplete ACL rupture can have subtle lameness, while dogs with complete ACL rupture have a weight-bearing pelvic limb lameness with possible external rotation of the affected limb (Tinga et al., 2018). Physical examination of dogs with ACL rupture may reveal atrophy of the pelvic limb musculature, knee effusion, medial periarticular fibrosis, and palpable anterior-posterior and internal rotational laxity (Muir 1997). Radiographs are a more sensitive diagnostic test than physical exam for detecting knee effusion and osteoarthritis associated with ACL rupture. Mild synovial effusion is an important sign of incomplete ACL rupture (Chuang et al., 2014). Environmental factors such as body weight and neutering influence disease risk. Although surgical and medical treatment options for ACL rupture exist there are no disease modifying treatments that prevent the development of knee osteoarthritis. Breeds at highest risk of ACL rupture include the Newfoundland, Rottweiler, Labrador Retriever, Boxer, and Bulldog (Witsberger et al., 2008).

**Supplementary References**

Bleedorn, J.A., Greuel, E.N., Manley, P.A., Schaefer, S.L., Markel, M.D., Holzman, G., et al. (2011). Synovitis in dogs with stable stifle joints and incipient cranial cruciate ligament rupture: a cross‐sectional study. *Vet Surg*. 40, 531-543. doi: 10.1111/j.1532-950X.2011.00841.x

Chuang, C., Ramaker, M.A., Kaur, S., Csomos, R.A., Kroner, K.T., Bleedorn, J.A., et al. (2014). Radiographic risk factors for contralateral rupture in dogs with unilateral cranial cruciate ligament rupture. *PLoS One*. 9, e106389. doi: 10.1371/journal.pone.0106389

Fels, L., Distl, O. (2014). Identification and validation of quantitative trait loci (QTL) for canine hip dysplasia (CHD) in German shepherd dogs. *PLoS One*. 9, e96618. doi: 10.1371/journal.pone.0096618

Ginja, M.M., Silvestre, A.M., Colaço, J., Gonzalo-Orden, J.M., Melo-Pinto, P., Orden, M.A., et al. (2009). Hip dysplasia in Estrela Mountain Dogs: prevalence and genetic trends 1991–2005. *Vet J*. 182, 275-282. doi: 10.1016/j.tvjl.2008.06.014

Ginja, M.M., Silvestre, A.M., Gonzalo-Orden, J.M., Ferreira, A.J. (2010). Diagnosis, genetic control and preventive management of canine hip dysplasia: a review. *Vet J*. 184, 269-276. doi: 10.1016/j.tvjl.2009.04.009

Hamann, H., Kirchhoff, T., Distl, O. (2003). Bayesian analysis of heritability of canine hip dysplasia in German Shepherd Dogs. *J Anim Breed Genet*. 120, 258-268. doi: 10.1046/j.1439-0388.2003.00395.x

Huang, M., Hayward, J.J., Corey, E., Garrison, S.J., Wagner, G.R., Krotscheck, U., et al. (2017). A novel iterative mixed model to remap three complex orthopedic traits in dogs. *PLoS One*. 12, e0176932. doi: 10.1371/journal.pone.0176932

Janutta, V., Hamann, H., Distl, O. (2006). Complex segregation analysis of canine hip dysplasia in German shepherd dogs. *Journal of Hered*. 97, 13-20. doi: 10.1093/jhered/esi128

King, M.D. (2017). Etiopathogenesis of canine hip dysplasia, prevalence, and genetics. *Vet Clin North Am Small Anim Pract*. 47, 753-767. doi: 10.1016/j.cvsm.2017.03.001

Krontveit, R.I., Nødtvedt, A., Sævik, B.K., Ropstad, E., Trangerud, C. (2012). Housing-and exercise-related risk factors associated with the development of hip dysplasia as determined by radiographic evaluation in a prospective cohort of Newfoundlands, Labrador Retrievers, Leonbergers, and Irish Wolfhounds in Norway. *Am J Vet Res*. 73, 838-846. doi: 10.2460/ajvr.73.6.838

Krotscheck, U., Todhunter, R.J. (2010). “Pathogenesis of hip dysplasia”, in Mechanisms of Disease in Small Animal Surgery 3rd Edition, eds. M.J. Bojrab, E. Monnet (Jackson WY, Teton NewMedia), 636-645.

Lust, G., Williams, A.J., Burton-Wurster, N., Pijanowski, G.J., Beck, K.A., Rubin, G., Smith, G.K. (1993). Joint laxity and its association with hip dysplasia in Labrador Retrievers. *Am J Vet Res*. 54, 1990-1999.

Mäki, K., Janss, L.L., Groen, A.F., Liinamo, A.E., Ojala, M. (2004). An indication of major genes affecting hip and elbow dysplasia in four Finnish dog populations. *Heredity*. 92, 402-408. doi: 10.1038/sj.hdy.6800434

Michelsen, J. (2013). Canine elbow dysplasia: aetiopathogenesis and current treatment recommendations. *Vet J*. 196, 12-19. doi: 10.1016/j.tvjl.2012.11.009

Muir, P. (1997). Physical examination of lame dogs. *Compendium on Continuing Education for the Practicing Veterinarian.* 19, 1149-1161.

Nap, R.C. (1995). Pathophysiology and clinical aspects of canine elbow dysplasia. *In Proceedings of the 7th International Elbow Working Group Meeting, Constance, Germany*, 6-8.

Pfahler, S., Distl, O. (2012). Identification of quantitative trait loci (QTL) for canine hip dysplasia and canine elbow dysplasia in Bernese Mountain Dogs. *PLoS One*. 7, e49782. doi: 10.1371/journal.pone.0049782

Richardson, D.C. (1992). The role of nutrition in canine hip dysplasia. *Vet Clin North Am Small Anim Pract*. 22, 529-540. doi: 10.1016/s0195-5616(92)50053-5

Sánchez-Molano, E., Woolliams, J.A., Pong-Wong, R., Clements, D.N., Blott, S.C., Wiener, P. (2014). Quantitative trait loci mapping for canine hip dysplasia and its related traits in UK Labrador Retrievers. *BMC Genomics*. 15, 833. doi: 10.1186/1471-2164-15-833

Smith, G.K. (1998). Canine hip dysplasia: pathogenesis, diagnosis, and genetic control. *Vet Q*. 20, S22-S24. doi: 10.1080/01652176.1998.10807390

Tinga, S., Kim, S.E., Banks, S.A., Jones, S.C., Park, B.H., Pozzi, A, et al. (2018). Femorotibial kinematics in dogs with cranial cruciate ligament insufficiency: a three-dimensional in-vivo fluoroscopic analysis during walking. *BMC Vet Res*. 14, 85. doi: 10.1186/s12917-018-1395-2

Todhunter, R.J., Bliss, S.P., Casella, G., Wu, R., Lust, G., Burton-Wurster, N.I., et al. (2003). Genetic structure of susceptibility traits for hip dysplasia and microsatellite informativeness of an outcrossed canine pedigree. *J Hered*. 94, 39-48. doi: 10.1093/jhered/esg006

Vermote, K.A., Bergenhuyzen, A.L., Gielen, I., van Bree, H., Duchateau, L., Van Ryssen, B. (2010). Elbow lameness in dogs of six years and older: arthroscopic and imaging findings of medial coronoid disease in 51 dogs. *Vet Comp Orthop Traumatol*. 23, 43-50. doi: 10.3415/VCOT-09-03-0032

Witsberger, T.H., Villamil, J.A., Schultz, L.G., Hahn, A.W., Cook, J.L. (2008). Prevalence of and risk factors for hip dysplasia and cranial cruciate ligament deficiency in dogs. *J Am Vet Med Assoc*. 232, 1818-1824. doi: 10.2460/javma.232.12.1818

Zhu, L., Zhang, Z., Friedenberg, S., Jung, S.W., Phavaphutanon, J., Vernier-Singer, M., et al. (2009). The long (and winding) road to gene discovery for canine hip dysplasia. *Vet J*. 181, 97-110. doi: 10.1016/j.tvjl.2009.02.008
